# RaCaT: An open source and easy to use radiomics calculator tool

**DOI:** 10.1371/journal.pone.0212223

**Published:** 2019-02-20

**Authors:** Elisabeth Pfaehler, Alex Zwanenburg, Johan R. de Jong, Ronald Boellaard

**Affiliations:** 1 Department of Nuclear Medicine and Molecular Imaging, University of Groningen, University Medical Center Groningen, Groningen, The Netherlands; 2 OncoRay–National Center for Radiation Research in Oncology, Faculty of Medicine and University Hospital Carl Gustav Carus, Technische Universität Dresden, Helmholtz-Zentrum Dresden—Rossendorf, Dresden, Germany; 3 National Center for Tumor Diseases (NCT), Partner Site Dresden, Germany: German Cancer Research Center (DKFZ), Heidelberg, Germany; 4 Faculty of Medicine and University Hospital Carl Gustav Carus, Technische Universität Dresden, Dresden, Germany; 5 Helmholtz Association / Helmholtz-Zentrum Dresden—Rossendorf (HZDR), Dresden, Germany; 6 German Cancer Consortium (DKTK), Partner Site Dresden, and German Cancer Research Center (DKFZ), Heidelberg, Germany; 7 Department of Radiology & Nuclear Medicine, Amsterdam University Medical Centers, Location VUMC, Amsterdam, The Netherlands; Beijing University of Technology, CHINA

## Abstract

**Purpose:**

The widely known field ‘Radiomics’ aims to provide an extensive image based phenotyping of e.g. tumors using a wide variety of feature values extracted from medical images. Therefore, it is of utmost importance that feature values calculated by different institutes follow the same feature definitions. For this purpose, the imaging biomarker standardization initiative (IBSI) provides detailed mathematical feature descriptions, as well as (mathematical) test phantoms and corresponding reference feature values. We present here an easy to use radiomic feature calculator, RaCaT, which provides the calculation of a large number of radiomic features for all kind of medical images which are in compliance with the standard.

**Methods:**

The calculator is implemented in C++ and comes as a standalone executable. Therefore, it can be easily integrated in any programming language, but can also be called from the command line. No programming skills are required to use the calculator. The software architecture is highly modularized so that it is easily extendible. The user can also download the source code, adapt it if needed and build the calculator from source. The calculated feature values are compliant with the ones provided by the IBSI standard. Source code, example files for the software configuration, and documentation can be found online on GitHub (https://github.com/ellipfaehlerUMCG/RaCat).

**Results:**

The comparison with the standard values shows that all calculated features as well as image preprocessing steps, comply with the IBSI standard. The performance is also demonstrated on clinical examples.

**Conclusions:**

The authors successfully implemented an easy to use Radiomics calculator that can be called from any programming language or from the command line. Image preprocessing and feature settings and calculations can be adjusted by the user.

## Introduction

Features describing image texture contain valuable information about important image characteristics and are applied in multiple disciplines. They can be used for object identification or the definition of region of interests (ROI) in e.g. radar or satellite images [1,2]. In medical images, textural information extracted from tumor regions has shown to provide valuable information about prognosis, tumor staging, and treatment response [3–5].

For this purpose, a large amount of imaging biomarkers is extracted from the tumor region and used for classification purposes. These feature values, also named radiomic features, include besides second-order textural features, shape, first-order statistical, and intensity-histogram based features. Radiomic features are used to build machine learning models which are e.g. used for prediction or classification [6,7]. However, until now, radiomic features are not used for clinical decision making as there is a lack of standardization in the majority of the steps in the radiomics pipeline.

One of these challenges is the lack of a standardized feature definition and calculation. Feature values reported by different institutions do not necessarily follow the same feature definition nor necessarily lead to identical results when used on the same images. This problem is aimed to be solved by the image biomarker standardization initiative (IBSI) by providing mathematical feature definitions and phantom data sets with corresponding feature values in order to standardize feature definitions and calculations [8,9]. Several open-source software packages, like LifeX, IBEX, CaPTK or CGITA, calculating radiomic features have been developed and published [10–14]. However, although this initiative is widely known, only few radiomic feature calculators are also standardizing the image preprocessing part of the radiomic pipeline, which is essential for feature calculations. Furthermore, in the majority of the software packages not all defined features are implemented.

In order to provide a feature calculator that comes with the correct feature implementation of all features defined by the IBSI standard, we developed a Radiomics calculator tool, RaCaT, that is easy to use and does not require any programming skills. We compare the feature values obtained by RaCaT with the feature values reported by IBSI. Moreover, some known feature values were extracted from phantom images and compared with the expected values.

## Materials and methods

### Description of the radiomics calculator

**Fig 1:**
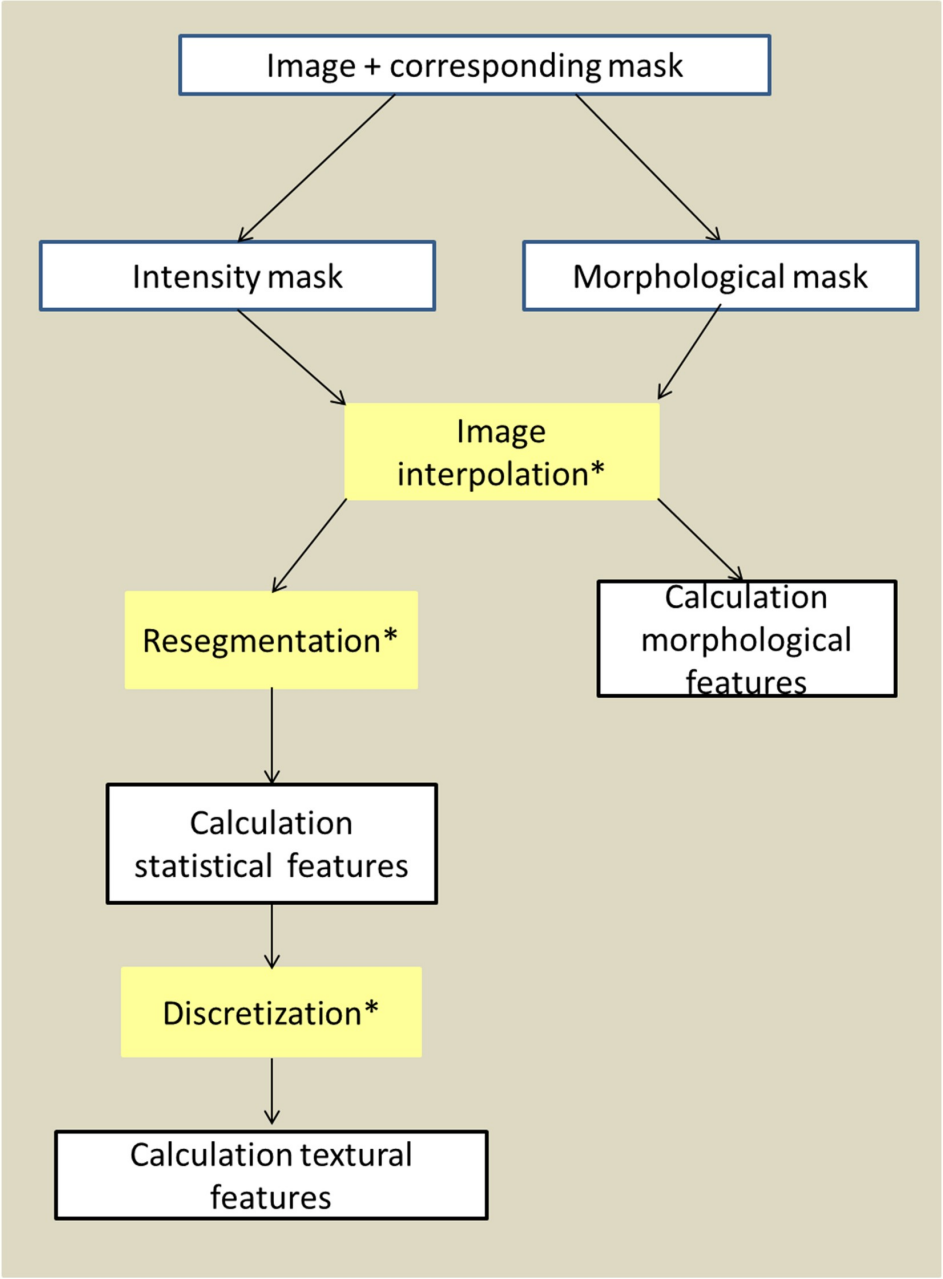
Workflow of RaCaT. All tasks marked with a * are optional and can be selected by the user.

Radiomics Calculator, RaCaT, calculates and returns a wide range of radiomic features for all kind of medical images. It is a standalone executable written in C++ that can be called from the command line but also from any programming language. It loads and preprocesses an input image and the corresponding mask, it calculates radiomic feature values and stores them in a user-defined output file. Furthermore, it stores the used preprocessing and feature calculation information in a separate file so that the user can easily track which settings were used for feature calculation. The workflow of RaCaT is illustrated in Fig 1.

### Installation/Compiling the calculator

In order to use RaCaT, visual C++ x86 has to be installed. The calculator is available in two ways: First, it can be downloaded as an executable that does not require any other library. The executable is available for Windows 32 and 64 bit and all Linux systems. Second, the source code of RaCaT is available and can be downloaded, modified if required, and be built from source. A precise description of the building process and which requirements have to be met can be found on GitHub (https://github.com/ellipfaehlerUMCG/RaCat).

### Implementation of the calculator

The implementation of RaCaT is highly modularized and therefore easily extendable. It consists of two basic classes and several feature group classes. The basic classes are used for reading and storing the information which is later passed to the feature group classes: One basic class reads and stores the parameters given in configuration files, necessary for image preprocessing, while the second class reads and preprocesses image and mask and stores the important image characteristics. All image preprocessing steps are implemented using the library Insight Toolkit (ITK) [13]. The following steps are implemented:

1. Image interpolation:
  The user can choose if the image should be interpolated using 3D or a slice-by-slice 2D interpolation. For both options, the image can be up or down sampled or it can be interpolated to isotropic voxels with 2 mm voxel size. The required interpolation algorithm can be set by the user (possibilities: trilinear, cubic spline or nearest neighbor interpolation).
2. Image discretization:
  Before the calculation of textural features, the image is usually discretized. Two discretization methods are implemented: a discretization with a fixed number of bins and a discretization with a fixed bin width. The number of bins as well as the bin width can be set by the user. Furthermore, the option to discretize the feature group intensity volume histogram separately is implemented.
3. Re-segmentation:
  In order to only include intensity values of a certain range in the volume-of-interest (VOI), the user can set a maximum and minimum intensity value that should be included in the VOI. Furthermore, RaCaT also supports the option to exclude outlier intensities of the VOI.

**Fig 2:**
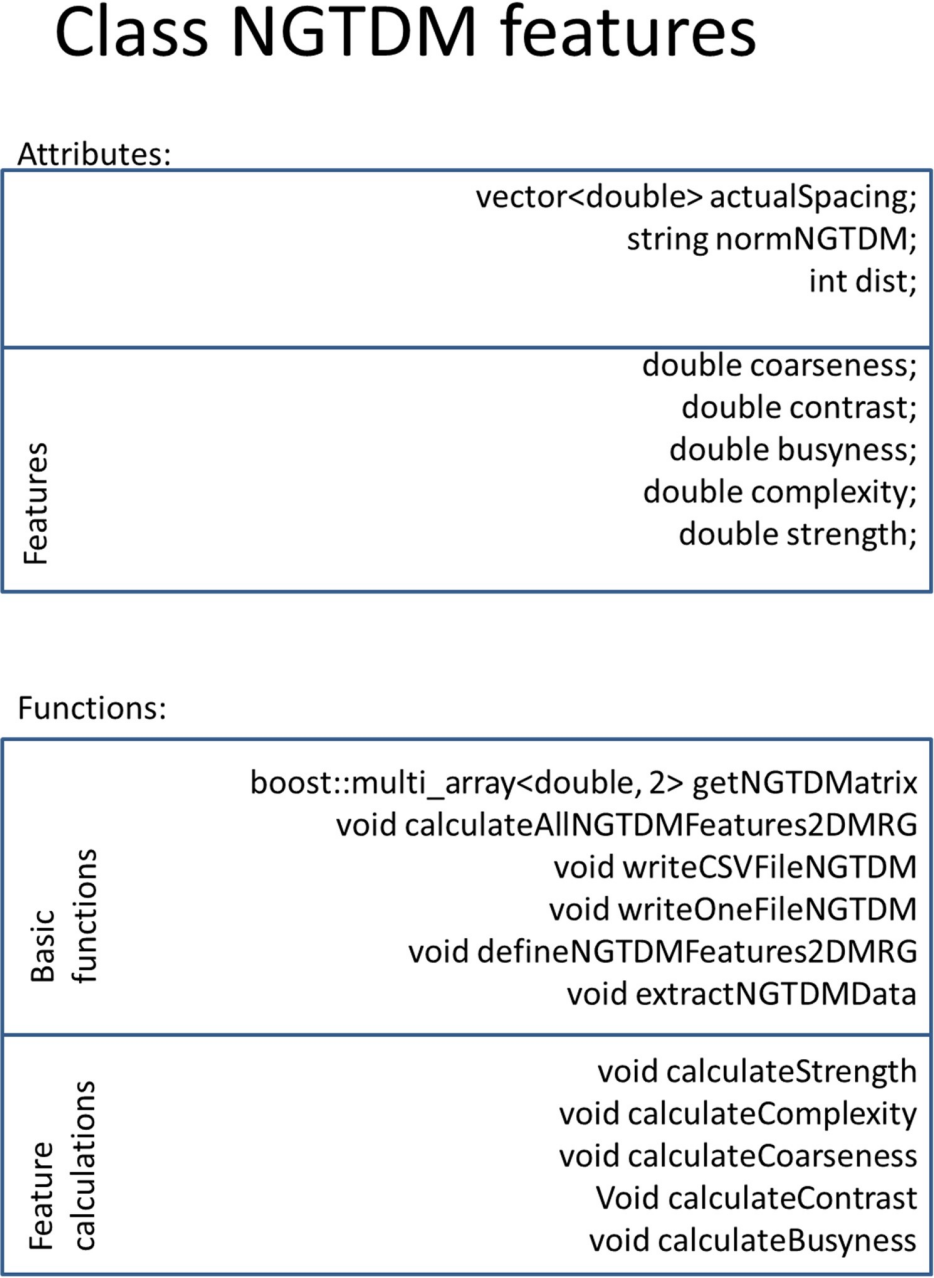
Organization of the class NGTDM features. The class has as attributes some basic values needed for the calculation of NGTDM features, as well as every NGTDM feature. The functions include the function to create the NGTD matrix, functions that fill and create the output file, and for every feature a function that calculates the feature.

Every feature group is realized with a separate class. If classes share feature calculations, the classes inherit from each other, but every feature group is independent and can be calculated separately. Every feature is calculated with a separate function. Therefore, additional feature calculations can be added easily. RaCaT is published under the BSD 3-Clause “New” or “Revised” License, what means that users do not have to submit their changes to the RaCaT repository, but that they have to mention the copyright of RaCaT when redistributing the code. Fig 2 displays as an example the implementation of the NGTDM feature class. The attributes of the class are the NGTDM features. For each of these features, a separate function is implemented assigning the feature value to the attribute. Furthermore, the class contains one separate method for the calculation of the NGTD matrix, as well as functions to fill and store the output files. All NGTDM feature groups inherit attributes and feature calculation functions from this class. While every NGTDM feature group calculates a different NGTD matrix and has separate functions to fill and write the output files. All other textural feature classes are implemented in the same way. A more detailed documentation of the code is available on GitHub.

### Documentation

The documentation is split in two parts: One part is written for users who are only interested in the use of the calculator. The second part explains more detailed the programming steps, lists classes and functions, and explains the heritages of the feature classes. Additionally, the code contains more comments which are not visible in the documentation.

### Usage of the calculator

In order to run RaCaT, some essential files have to be provided to the software which are described in more detail below. The locations of these files have to be given as parameter to the executable, accompanied by specific abbreviations. All required files including the abbreviations are listed in Table 1.

**Table 1:** Files required by RaCaT including their abbreviations that have to be given to the executable.

Abbreviation	Parameter	
- -ini	C:/RadiomicsTool/config.ini	Path to configuration file, where preprocessing steps and settings can be set
—img	C:/RadiomicsTool/*image*.nii	Path to image file. *Image* can be any filename If the *image* is in DICOM format, every image series should be stored in a separate folder. The path of this folder has to be given as parameter.
—voi if voi is not RT struct	C:/RadiomicsTool/*voi*.nii	Path to VOI. *VOI* can be any file name. If *VOI* is in DICOM format, path to folder containing the dicom series has to be given
—rts if voi is RT struct	C:/RadiomicsTool/RS_image.dcm	Path to VOI, if VOI is RT struct. RS_image can be any filename.
—out	C:/RadiomicsTool/output	Path to desired output. The output file is generated automatically and ‘.csv’ is automatically added to the name. If the file already exists, date and time of the calculations are added to the original name and the feature values are saved under this new name.
—pat	C:/RadiomicsTool/patientInfo.ini	*Only* for PET images: path to patient info file, containing necessary patient demographics and scan information required for SUV scaling. The user should generate and populate this file.
—fod	C:/RadiomicsTool/featuredefinition.ini	*Optional*: path to featuredefinition.ini, where the user can specify which feature groups should be calculated

**Fig 3:**
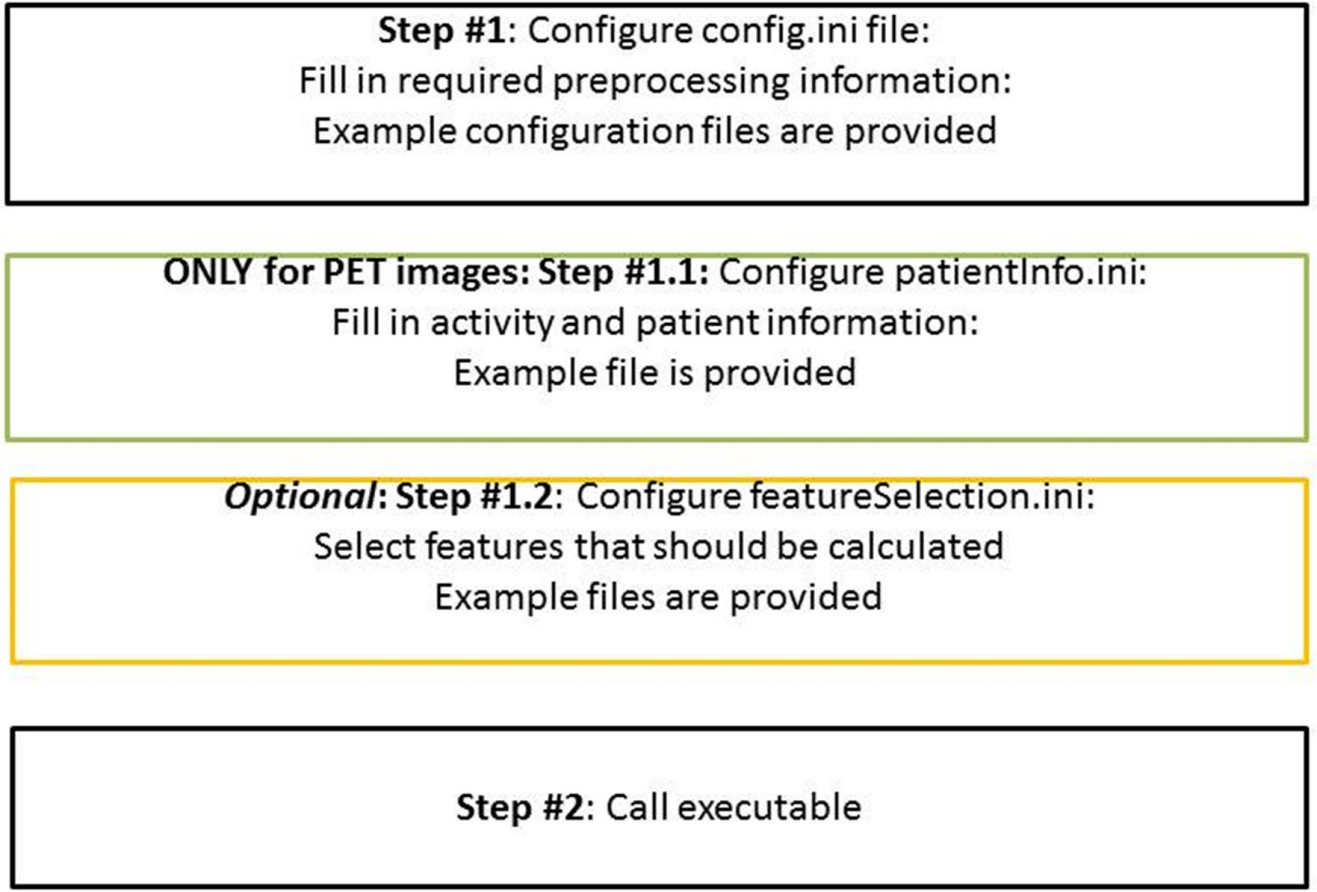
Necessary steps for running the executable.

Fig 3 illustrates the steps which have to be performed before the feature calculation starts:

- First, a configuration file has to be modified: here the desired preprocessing steps can be specified. If the same preprocessing steps are used for several images, the configuration file can be reused and has to be changed only once in the beginning.
- Second, if the input image is a PET image, also a patient information file has to be provided. This patient information file contains all important parameters regarding patient demographics and PET study information required to apply scaling of image intensities (activity concentration in Bq/mL) to SUV.
- Third, the user can optionally select only certain features for calculation. He can do this by adapting a feature output definition file.

Examples of frequently used configuration and feature output definition files as well as a patient information file can be found on GitHub. Furthermore, example commands how to call the executable with different image types can be found in the supplemental (S1 Fig).

### Feature calculation

RaCaT contains ten feature groups: morphological features providing information about tumor shape, a group of first-order statistical features, statistical intensity histogram features, intensity volume features and local intensity features. Furthermore, the following textural feature groups are implemented: grey-level co-occurrence matrices (GLCM) [14], grey-level run-length matrices (GLRLM) [15], grey-level size zone matrices (GLSZM) [16], grey-level distance zone matrices (GLDZM) [17], neighborhood-grey-tone difference matrices (NGTDM) [2] and neighborhood-grey-level dependence matrices (NGLDM) [18] (see Table 2). First-order, morphological and local intensity features are calculated before discretization. All other feature groups are calculated after image discretization. All textural features can be calculated slice by slice (2D) and by including the whole volume (3D). For both dimensions, different ways to merge texture matrices and features are implemented. This includes the following options:

- For each 2D directional matrix, features are calculated and then averaged over the 2D directions and slices
- 2D directional matrices are first merged per slice, then features are extracted from this matrix
- The 2D directional matrices are merged per direction and then the average of each direction matrix is calculated. From this matrix, features are extracted.
- Before feature calculation, all 2D directional matrices are merged.
- Features are extracted from each 3D directional matrix. These features are averaged over directions.
- Before feature calculation, all 3D directional matrices are merged.

**Table 2:** Implemented feature groups and corresponding abbreviations.

- Feature class	Feature group	Abbreviation
	Morphological features	
	Statistical features	
Intensity histogram features	Intensity histogram features	IH
	Intensity volume histogram features	IVH
Textural features	Grey-level-co-occurrence matrix	GLCM
	Grey-level-run-length matrix	GLRLM
	Grey-level-size-zone matrix	GLSZM
	Grey-level-distance-zone matrix	GLDZM
	Neighborhood-grey-tone-difference matrix	NGTDM
	Neighborhood-grey-level-dependence matrix	NGLDM

### Required input files

#### Image and VOI

An image and a corresponding image mask (or VOI) are required as input for the calculator. Mask and image should be aligned and have the same dimensions. The mask can either be provided as binary mask with any constant value marking the VOI (usually 1) or the VOI can be marked by intensity values of a certain range. In this case, the user can set the threshold value up to which percentage of the maximum value the voxels should be included in the mask. This can be done by changing the parameter ThresholdForVOI in the configuration file. Mask and image can be given in one of the following formats: nrrd, nifti, DICOM, analyze, as well as raw data. The mask can also be given as a radiotherapy (RT)-struct. If the mask is given as RT-struct, the command to call the executable is slightly different from the call used for the other formats (see Table 1). If image or mask are in DICOM format, it is important that every DICOM image series is stored in a separate folder. The name of this folder has then given to the executable (compare also with the example commands provided in the supplemental S1 Fig). In one run, RaCaT calculates the radiomic features for one image and mask. It is not possible to calculate radiomic features for several images at once. However, an example of a Python script, calling RaCaT for several images and masks is available in GitHub material.

#### Configuration file

**Fig 4:**
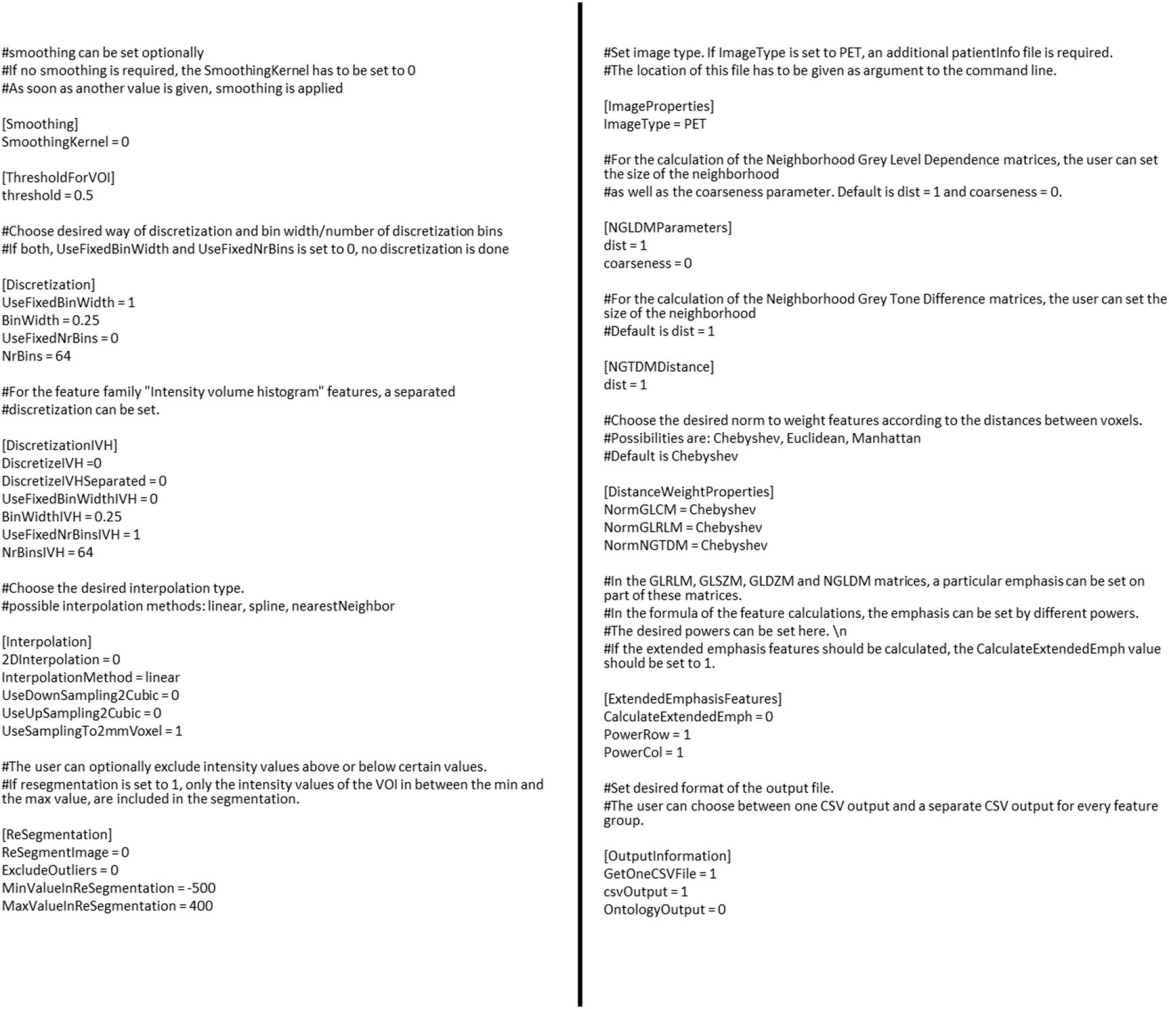
Example of configuration file. The user can set the required preprocessing steps, like e.g. re-segmentation by setting the ReSegmentImage parameter from 0 to 1. Other parameters like the number of bind (NrBins) can also be set to any required value.

In a config.ini file, the user can select the preprocessing steps that are performed before the feature calculation starts. An example for a config.ini file is displayed in Fig 4. More examples of the config.ini file including the most common used preprocessing steps, can be found in GitHub. If the user wants to calculate radiomic features for several bin width or number of bins, a separate configuration file has to be created for every configuration. As this can be time consuming, a python script how to create several configuration files with different number of bins as well as a script calling the RaCaT executable several times with different configuration files is available.

#### Additional file for PET images

If the image is a PET image, the program converts the intensity values from Bq/ml to SUV (Standardized Uptake Value) or SUL (Standardized Uptake value normalized to lean body mass). Here fore, some patient characteristics (weight, height, gender) as well as the net injected activity and injection time are required. Furthermore, the user has the possibility to set a scaling parameter. If this scaling parameter is set, all other values are ignored and every image intensity value is simply multiplied with this scaling parameter.

#### Feature output definition file

**Fig 5:**
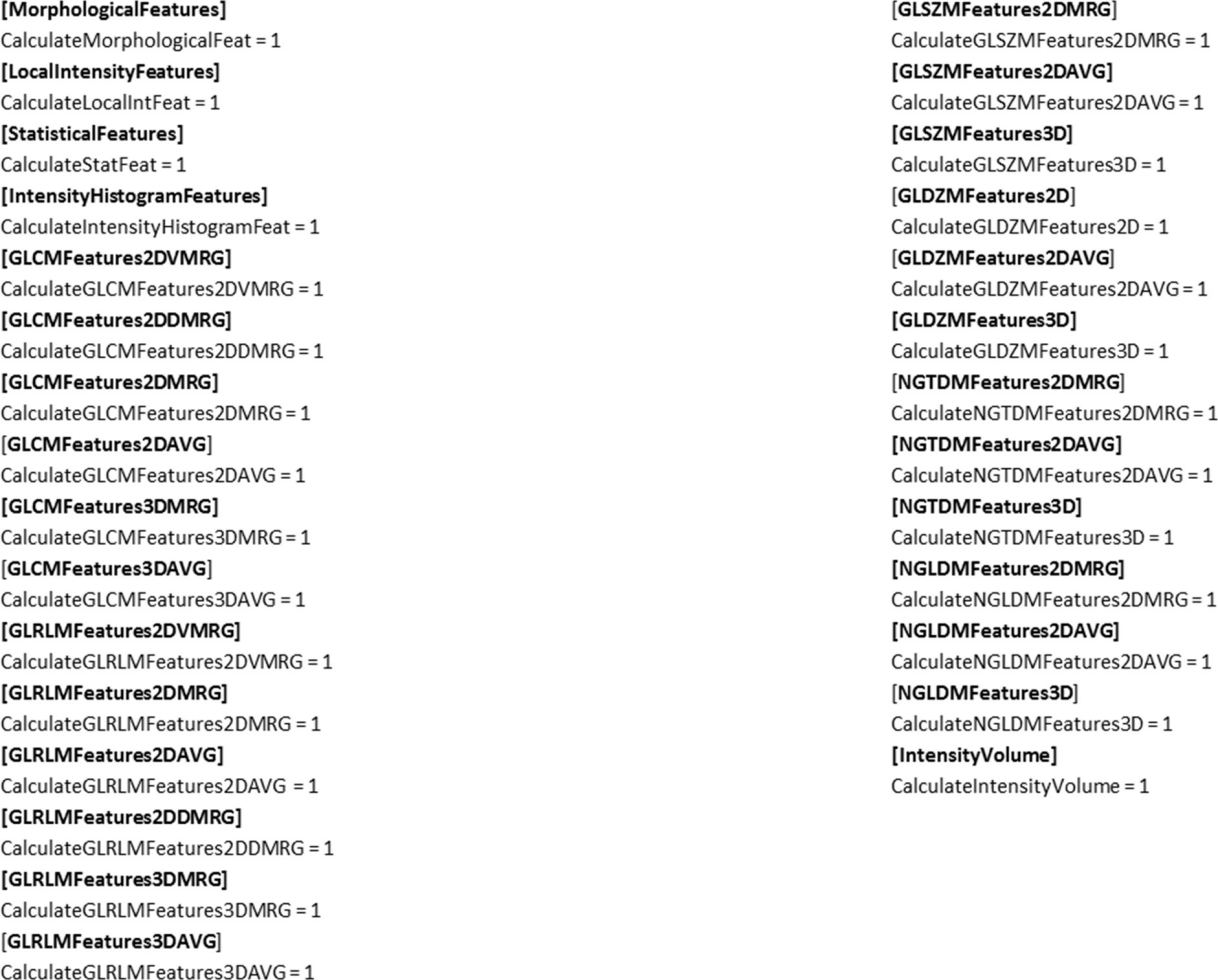
Example of feature output definition file. If a feature group should not be included in the calculations, the value for the corresponding group has to be set to 0.

Furthermore, the user has the option to select only certain feature groups he wants to include in the calculation. This can be done in a separate file called featureOutputDefinition.ini. This is optional. The location of the feature output definition file has to be given as parameter to the calculator. If no feature output definition file is given, all available features are calculated. An example of a feature output definition file is displayed in Fig 5.

#### Output files

The calculated feature values are stored with floating point precision in one or more comma-separated-value (csv) files. The feature names which are listed in the output files are the names proposed by the IBSI standard. To ease a further documentation, two additional output files are created: The first output is a copy of the used configuration file so that the user can easily access which preprocessing steps were included in the feature calculation. The second output file contains information about the input images and calculated feature groups. The filenames of all output files are aligned so that the user can easily track which output files are belonging to one calculation step.

### Testing

To ensure that the toolbox calculates values compliant with the standard, the calculated feature values were compared with the IBSI standard. The initiative provides two phantoms that can be used for comparison: one small, artificial mathematical phantom and a CT-image with a corresponding RT-struct of the VOI. For the CT-image, several configurations with variations in discretization, resegmentation, and interpolation method are available for comparison. In order to validate RaCaT, both phantoms have been used for comparison. The calculated feature values, as well as the corresponding IBSI values are listed in supplemental S1 Table, S2 Table, and S3 Table. For every feature value, IBSI provides tolerance levels depending on the used configuration. As can be seen, almost all feature values are in the provided tolerance levels. Only for morphological features, small deviations were found. Here, the volume differed from 0.2% - 1% from the volume given by the IBSI standard, while the surface had a deviation from 2%-10%. Therefore, all morphological features which are dependent of surface and volume also show slight deviations.

**Fig 6:**
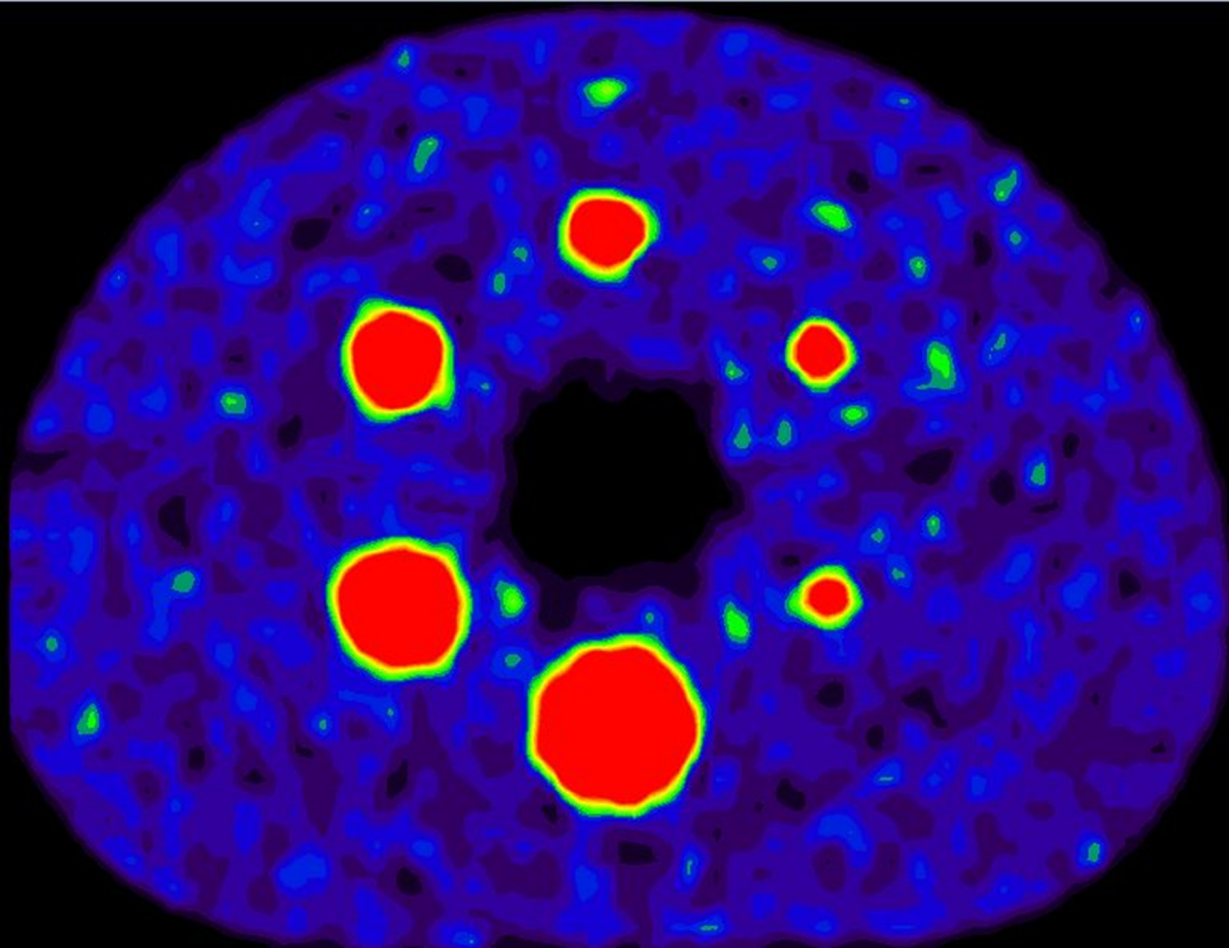
PET Scan of the NEMA image quality phantom. The image quality phantom contains six spheres with different diameters. For comparison, the spheres were segmented in the image and morphological features were calculated.

Therefore, morphological feature values were further compared with values obtained from a phantom scan. For this purpose, a positron emission tomography combined with computed tomography (PET/CT) scan of the NEMA image quality phantom was acquired on a Siemens Biograph mCT64 (Siemens Healthcare, Knoxville, USA) (see Fig 6). The NEMA image quality phantom consists of six spheres with diameters 37, 28, 21, 17, 13, and 10 mm which are placed in a large background compartment. Spheres were filled with a fluorodeoxyglucose (FDG) activity solution of 19.76 kBq/ml, while the background was filled with 1.94 kBq/ml, so that a sphere-to-background ratio of around 10:1 was obtained. The image was reconstructed to a voxel size of 3.1819 x 3.1819 x 2 mm using the vendor provided PSF+TOF reconstruction method with three iterations and 21 subsets (PSFTOF 3i21s). The spheres were manually delineated in the images by placing a sphere with the exact diameter on the right position in the images. Consequently, the correct shape feature values are known and can be used for comparison with the feature values calculated by RaCaT. The expected and calculated feature values are listed in Table 3. The comparison showed that for the bigger spheres (diameter 37–17 mm) the percentage deviation between calculated and expected shape feature values differs from 1–10%, with 93.75% of the features showing a deviation less than 5% (see Table 3). For the smaller spheres (13 mm and 10 mm), the deviation increased to 1–19%.

**Table 3:** Morphological features calculated by RaCaT, the expected value, and the percentage differences between these two values for the spheres of the NEMA image quality phantom.

		Tool	Expected value	Percentage difference (%)
Sphere1	Volume	25898.4	26521	2.35
	Surface	4194.92	4300	2.44
	maximum 3D diameter	36.7149	37	0.77
	Sphericity	1.00912	1	0.91
Sphere2	Volume	11035.7	11494	3.99
	Surface	2413.78	2463	2.00
	maximum 3D diameter	0.993085	1	0.69
	Sphericity	27.6493	28	1.25
Sphere3	Volume	5811.46	5575	4.24
	Surface	1542.14	1521	1.39
	maximum 3D diameter	1.01364	1	1.36
	Sphericity	21.6559	22	1.56
Sphere4	Volume	2855.11	2572	11.01
	Surface	952.166	907	4.98
	maximum 3D diameter	1.02216	1	2.22
	Sphericity	17.4926	18	2.82
Sphere5	Volume	951.702	1150	17.24
	Surface	452.697	530	14.59
	maximum 3D diameter	12.0414	12	0.34
	Sphericity	1.03358	1	3.36
Sphere6	Volume	425	523	18.74
	Surface	259	314	17.52
	maximum 3D diameter	9	10	10.00
	Sphericity	1.05	1	5.00

### Application to clinical data

**Fig 7:**
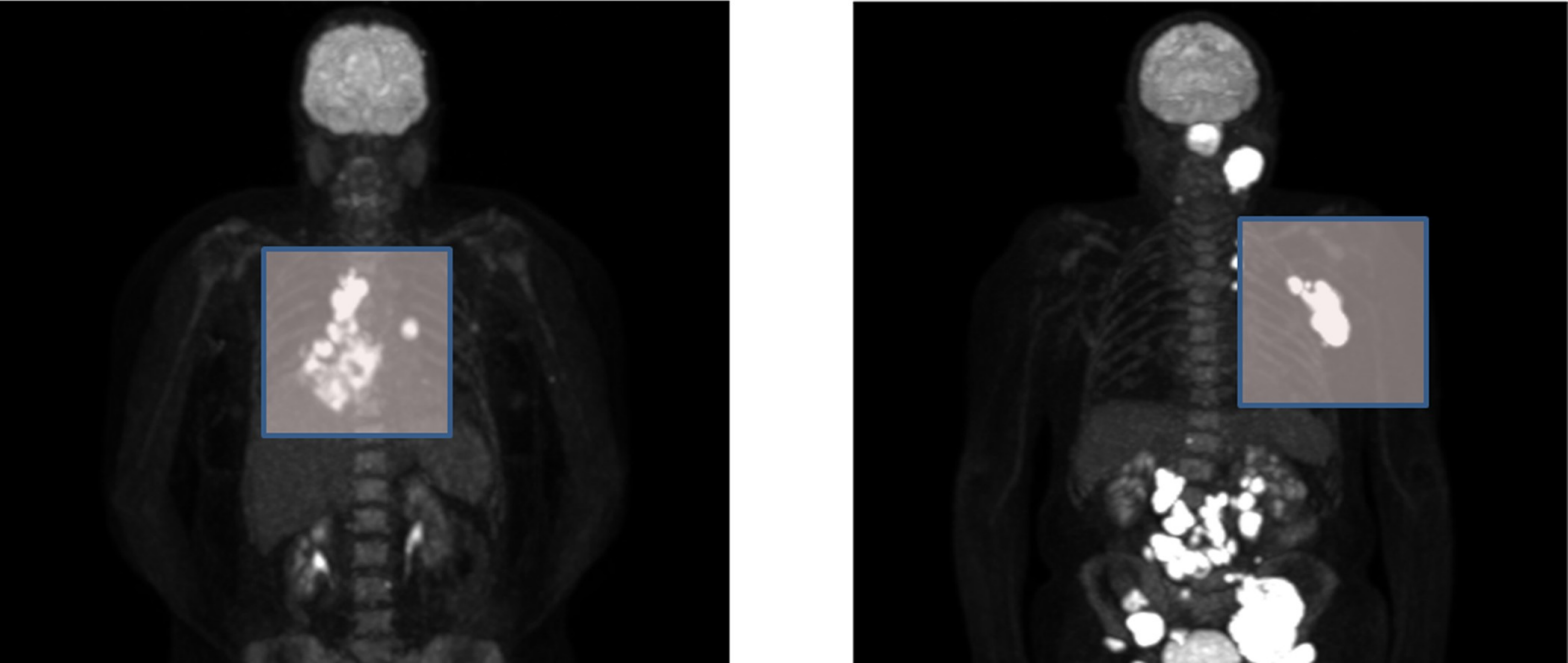
Maximum intensity projection of Patient 1 (left) and patient 2 (right). The tumors used for feature calculation are marked in the images. Tumors were manually segmented and used for computation of radiomic features.

Moreover, radiomic features were extracted from two PET-images of cancer patients. Both patients were scanned on a Siemens Biograph mCT64, and the images were iteratively reconstructed using the PSF+TOF reconstruction method (PSF+TOF 3i21s) implemented in the scanner and a post-reconstruction smoothing with a 6.5 mm full-width-at-half-maximum Gaussian kernel. Images were reconstructed to a voxel size of 3.1819 mm x 3.1819 mm x 2 mm. Patient 1 was injected with 245 MBq 85 minutes before scan start, while patient 2 was injected with 229 MBq 60 minutes before scan start. Maximum intensity projection images of both patients are displayed in Fig 7. Tumors were manually delineated by an experienced radiologist. All implemented features were calculated by RaCaT and are listed in Table 4. As can be seen, feature values are changing as a function of the tumors.

**Table 4:** Radiomic features extracted from cancer patients.

		Melanoma 2	Melanoma 3
Morphology	Volume	132550	30353.2
Morphology	approximate volume	132550	30353.2
Morphology	Surface	18076.9	7522.4
Morphology	Surface to volume ratio	0.136378	0.247829
Morphology	Compactness1	0.0307693	0.026248
Morphology	Compactness2	0.336386	0.244789
Morphology	Spherical disproportion	1.43787	1.59858
Morphology	sphericity	0.695472	0.625553
Morphology	asphericity	0.437869	0.598578
Morphology	center of mass shift	46.4677	57.5868
Morphology	maximum 3D diameter	115.629	79.2605
Morphology	major axis length	89.949	77.541
Morphology	minor axis length	73.687	36.5853
Morphology	least axis length	35.6244	21.6824
Morphology	elongation	0.905101	0.686891
Morphology	flatness	0.629326	0.528796
Morphology	vol density AABB	0.223048	0.196102
Morphology	area density AABB	0.424074	0.421014
Morphology	vol density AEE	1.07213	0.942456
Morphology	integrated intensity	2.15E+06	412930
Morphology	Morans I	0.13243	0.086137
Morphology	Gearys C	1.03999	0.943915
Local intensity	local intensity peak	63966.5	30248
Local intensity	global intensity peak	64101	32327.2
Statistics	mean	16.2486	13.6041
Statistics	variance	49.4711	11.0447
Statistics	skewness	0.470834	0.420448
Statistics	kurtosis	-0.760356	-0.94058
Statistics	median	15.1567	13.1035
Statistics	minimum	6.12322	8.83092
Statistics	10th percentile	7.65485	9.54748
Statistics	90th percentile	25.898	18.4434
Statistics	maximum	35.8524	21.5399
Statistics	Interquartile range	11.4069	5.48123
Statistics	range	29.7292	12.709
Statistics	Mean absolut deviation	5.95899	2.8419
Statistics	Robust mean absolute deviation	4.73687	2.33481
Statistics	Median absolute deviation	5.90466	2.81521
Statistics	Coefficient of variation	0.432873	0.24429
Statistics	Quartile coefficient	0.359105	0.204447
Statistics	Energy	2.05E+06	293981
Statistics	Root mean	17.7056	14.0042
intensity volume	volume at int fraction 10	0.758326	0.712475
intensity volume	volume at int fraction 90	0.00824931	0.003336
intensity volume	int at vol fraction 10	27.1232	19.8309
intensity volume	int at vol fraction 90	8.12322	10.8309
intensity volume	difference vol at int fraction	0.750076	0.709139
intensity volume	difference int at volume fraction	19	9
Intensity histogram	mean	40.9937	19.6191
Intensity histogram	variance	791.594	176.755
Intensity histogram	skewness	0.470703	0.421877
Intensity histogram	kurtosis	-0.761046	-0.94283
Intensity histogram	median	37	18
Intensity histogram	minimum	1	1
Intensity histogram	10th percentile	7	3
Intensity histogram	90th percentile	80	39
Intensity histogram	maximum	119	51
Intensity histogram	mode	2	1
Intensity histogram	Interquartile range	45	22
Intensity histogram	range	118	50
Intensity histogram	Mean absolut deviation	23.8357	11.3695
Intensity histogram	Robust mean absolute deviation	18.6017	9.22622
Intensity histogram	Median absolut deviation	23.6195	11.2628
Intensity histogram	Coefficient of variation	0.686331	0.677653
Intensity histogram	Quartile coefficient	0.56962	0.578947
Intensity histogram	Entropy	6.61231	5.49251
Intensity histogram	Uniformity	0.0111764	0.024041
Intensity histogram	Energy	1.62E+07	841933
Intensity histogram	Maximum histogram gradient	14	8.5
Intensity histogram	Maximum histogram gradient grey level	56	15
Intensity histogram	Minimum histogram gradient	-12	-19
Intensity histogram	Minimum histogram gradient grey level	32	1
glcmFeatures2Davg	joint maximum	0.0202253	0.061685
glcmFeatures2Davg	joint average	40.4281	18.3173
glcmFeatures2Davg	joint variance	551.638	104.981
glcmFeatures2Davg	joint entropy	7.47154	5.15897
glcmFeatures2Davg	difference average	13.6741	9.01583
glcmFeatures2Davg	difference variance	112.914	39.6169
glcmFeatures2Davg	difference entropy	4.61465	3.37
glcmFeatures2Davg	sum average	80.8563	36.6345
glcmFeatures2Davg	sum variance	1889.54	286.275
glcmFeatures2Davg	sum entropy	5.9103	3.9222
glcmFeatures2Davg	angular second moment	0.0114301	0.048791
glcmFeatures2Davg	contrast	317.009	133.648
glcmFeatures2Davg	dissimilarity	13.6741	9.01583
glcmFeatures2Davg	inverse difference	0.156578	0.173049
glcmFeatures2Davg	inverse difference normalised	0.903083	0.822705
glcmFeatures2Davg	inverse difference moment	0.0856765	0.092541
glcmFeatures2Davg	inverse difference moment normalised	0.979129	0.91682
glcmFeatures2Davg	inverse variance	0.0922006	0.100673
glcmFeatures2Davg	correlation	0.630216	0.239559
glcmFeatures2Davg	autocorrelation	2215.83	442.887
glcmFeatures2Davg	cluster tendency	1889.54	286.275
glcmFeatures2Davg	cluster shade	14816.1	160.837
glcmFeatures2Davg	cluster prominence	9.05E+06	244779
glcmFeatures2Davg	first measure of information correlation	-0.664322	-0.72547
glcmFeatures2Davg	second measure of information correlation	0.998302	0.955912
glcmFeatures2DDmrg	joint maximum	0.0011916	0.00301
glcmFeatures2DDmrg	joint average	45.5856	22.8963
glcmFeatures2DDmrg	joint variance	749.941	175.007
glcmFeatures2DDmrg	joint entropy	12.0046	10.1074
glcmFeatures2DDmrg	difference average	13.8464	10.4439
glcmFeatures2DDmrg	difference variance	128.924	54.4645
glcmFeatures2DDmrg	difference entropy	5.16868	4.67357
glcmFeatures2DDmrg	sum average	91.1712	45.7926
glcmFeatures2DDmrg	sum variance	2671.56	532.294
glcmFeatures2DDmrg	sum entropy	7.5715	6.40143
glcmFeatures2DDmrg	angular second moment	0.000292373	0.001045
glcmFeatures2DDmrg	contrast	328.216	167.736
glcmFeatures2DDmrg	dissimilarity	13.8464	10.4439
glcmFeatures2DDmrg	inverse difference	0.154703	0.166918
glcmFeatures2DDmrg	inverse difference normalised	0.902181	0.842111
glcmFeatures2DDmrg	inverse difference moment	0.0829486	0.086833
glcmFeatures2DDmrg	inverse difference moment normalised	0.978452	0.944593
glcmFeatures2DDmrg	inverse variance	0.087643	0.091594
glcmFeatures2DDmrg	correlation	0.781185	0.526972
glcmFeatures2DDmrg	autocorrelation	2664.74	615.467
glcmFeatures2DDmrg	cluster tendency	2671.55	532.294
glcmFeatures2DDmrg	cluster shade	60986.8	2780.95
glcmFeatures2DDmrg	cluster prominence	1.64E+07	588513
glcmFeatures2DDmrg	first measure of information correlation	-0.199431	-0.18583
glcmFeatures2DDmrg	second measure of information correlation	0.962546	0.934605
glcmFeatures2Dmrg	joint maximum	0.010219	0.028869
glcmFeatures2Dmrg	joint average	40.3951	18.3675
glcmFeatures2Dmrg	joint variance	552.561	105.171
glcmFeatures2Dmrg	joint entropy	9.12932	6.81417
glcmFeatures2Dmrg	difference average	13.5621	8.9456
glcmFeatures2Dmrg	difference variance	119.146	42.6295
glcmFeatures2Dmrg	difference entropy	4.97349	4.02724
glcmFeatures2Dmrg	sum average	80.7901	36.7351
glcmFeatures2Dmrg	sum variance	1897.84	290.538
glcmFeatures2Dmrg	sum entropy	6.7243	5.02938
glcmFeatures2Dmrg	angular second moment	0.00424268	0.018702
glcmFeatures2Dmrg	contrast	312.403	130.146
glcmFeatures2Dmrg	dissimilarity	13.5621	8.9456
glcmFeatures2Dmrg	inverse difference	0.157532	0.17777
glcmFeatures2Dmrg	inverse difference normalised	0.90378	0.836062
glcmFeatures2Dmrg	inverse difference moment	0.0863837	0.095707
glcmFeatures2Dmrg	inverse difference moment normalised	0.979421	0.930654
glcmFeatures2Dmrg	inverse variance	0.0929435	0.105291
glcmFeatures2Dmrg	correlation	0.636783	0.266349
glcmFeatures2Dmrg	autocorrelation	2215.28	444.145
glcmFeatures2Dmrg	cluster tendency	1897.84	290.538
glcmFeatures2Dmrg	cluster shade	14138.1	112.888
glcmFeatures2Dmrg	cluster prominence	9.13E+06	249776
glcmFeatures2Dmrg	first measure of information correlation	-0.361666	-0.31522
glcmFeatures2Dmrg	second measure of information correlation	0.978974	0.92285
glcmFeatures2Dvmrg	joint maximum	0.0010181	0.002227
glcmFeatures2Dvmrg	joint average	45.551	22.8755
glcmFeatures2Dvmrg	joint variance	750.815	174.593
glcmFeatures2Dvmrg	joint entropy	12.4418	10.7059
glcmFeatures2Dvmrg	difference average	13.7442	10.2998
glcmFeatures2Dvmrg	difference variance	135.013	56.9565
glcmFeatures2Dvmrg	difference entropy	5.22235	4.74088
glcmFeatures2Dvmrg	sum average	91.1019	45.751
glcmFeatures2Dvmrg	sum variance	2679.36	535.329
glcmFeatures2Dvmrg	sum entropy	7.59971	6.45314
glcmFeatures2Dvmrg	angular second moment	0.000219332	0.00069
glcmFeatures2Dvmrg	contrast	323.917	163.041
glcmFeatures2Dvmrg	dissimilarity	13.744	10.2998
glcmFeatures2Dvmrg	inverse difference	0.155523	0.168325
glcmFeatures2Dvmrg	inverse difference normalised	0.902821	0.843792
glcmFeatures2Dvmrg	inverse difference moment	0.0835302	0.087826
glcmFeatures2Dvmrg	inverse difference moment normalised	0.978729	0.945953
glcmFeatures2Dvmrg	inverse variance	0.0882732	0.092581
glcmFeatures2Dvmrg	correlation	0.784291	0.533079
glcmFeatures2Dvmrg	autocorrelation	2663.74	616.36
glcmFeatures2Dvmrg	cluster tendency	2679.35	535.329
glcmFeatures2Dvmrg	cluster shade	60503.1	2787.42
glcmFeatures2Dvmrg	cluster prominence	1.65E+07	592233
glcmFeatures2Dvmrg	first measure of information correlation	-0.134096	-0.07906
glcmFeatures2Dvmrg	second measure of information correlation	0.912545	0.765345
glcmFeatures3Davg	joint maximun	0.00127655	0.003497
glcmFeatures3Davg	joint average	45.5488	22.8304
glcmFeatures3Davg	joint variance	752.246	175.424
glcmFeatures3Davg	joint entropy	11.9768	10.0788
glcmFeatures3Davg	difference average	13.7407	10.2897
glcmFeatures3Davg	difference variance	133.057	55.5321
glcmFeatures3Davg	difference entropy	5.14368	4.65039
glcmFeatures3Davg	sum average	91.0975	45.6608
glcmFeatures3Davg	sum variance	2675.58	533.909
glcmFeatures3Davg	sum entropy	7.57145	6.40178
glcmFeatures3Davg	angular second moment	0.000302383	0.001075
glcmFeatures3Davg	contrast	333.4	167.789
glcmFeatures3Davg	dissimilarity	13.7407	10.2897
glcmFeatures3Davg	inverse difference	0.159499	0.175979
glcmFeatures3Davg	inverse difference normalised	0.903224	0.844849
glcmFeatures3Davg	inverse difference moment	0.0870708	0.096589
glcmFeatures3Davg	inverse difference moment normalised	0.978248	0.944981
glcmFeatures3Davg	inverse variance	0.0900469	0.101335
glcmFeatures3Davg	correlation	0.778516	0.528182
glcmFeatures3Davg	autocorrelation	2661.41	613.059
glcmFeatures3Davg	cluster tendency	2675.58	533.909
glcmFeatures3Davg	cluster shade	60809.1	2814.04
glcmFeatures3Davg	cluster prominence	1.65E+07	594057
glcmFeatures3Davg	first measure of information correlation	-0.203614	-0.1904
glcmFeatures3Davg	second measure of information correlation	0.963043	0.936539
glcmFeatures3DWmrg	joint maximun	0.00102245	0.001574
glcmFeatures3DWmrg	joint average	45.5004	22.7831
glcmFeatures3DWmrg	joint variance	753.414	175.218
glcmFeatures3DWmrg	joint entropy	12.5416	10.8285
glcmFeatures3DWmrg	difference average	13.5902	10.0637
glcmFeatures3DWmrg	difference variance	142.512	59.9825
glcmFeatures3DWmrg	difference entropy	5.22012	4.74307
glcmFeatures3DWmrg	sum average	91.0008	45.5663
glcmFeatures3DWmrg	sum variance	2686.45	539.612
glcmFeatures3DWmrg	sum entropy	7.6062	6.46966
glcmFeatures3DWmrg	angular second moment	0.000208636	0.000632
glcmFeatures3DWmrg	contrast	327.204	161.26
glcmFeatures3DWmrg	dissimilarity	13.5902	10.0637
glcmFeatures3DWmrg	inverse difference	0.160884	0.179246
glcmFeatures3DWmrg	inverse difference normalised	0.90416	0.847648
glcmFeatures3DWmrg	inverse difference moment	0.0881152	0.099262
glcmFeatures3DWmrg	inverse difference moment normalised	0.978639	0.94692
glcmFeatures3DWmrg	inverse variance	0.0911324	0.103955
glcmFeatures3DWmrg	correlation	0.782853	0.53983
glcmFeatures3DWmrg	autocorrelation	2660.1	613.66
glcmFeatures3DWmrg	cluster tendency	2686.45	539.612
glcmFeatures3DWmrg	cluster shade	60150	2769.21
glcmFeatures3DWmrg	cluster prominence	1.66E+07	601776
glcmFeatures3DWmrg	first measure of information correlation	-0.119212	-0.05671
glcmFeatures3DWmrg	second measure of information correlation	0.892214	0.684467
GLRLMFeatures2Davg	short run emphasis	0.982296	0.984213
GLRLMFeatures2Davg	long runs emphasis	1.07575	1.06635
GLRLMFeatures2Davg	Low grey level run emphasis	0.0324134	0.094494
GLRLMFeatures2Davg	High grey level run emphasis	2054.96	419.531
GLRLMFeatures2Davg	Short run low grey level emphasis	0.0317711	0.093822
GLRLMFeatures2Davg	Short run high grey level emphasis	2004.49	410.815
GLRLMFeatures2Davg	Long run low grey level emphasis	0.0351005	0.097206
GLRLMFeatures2Davg	Long run high grey level emphasis	2272.94	456.173
GLRLMFeatures2Davg	Grey level non uniformity	2.76352	1.91514
GLRLMFeatures2Davg	Grey level non uniformity normalized	0.0311729	0.103436
GLRLMFeatures2Davg	Run length non uniformity	138.548	36.1029
GLRLMFeatures2Davg	Run length non uniformity normalized	0.955012	0.961134
GLRLMFeatures2Davg	Run percentage	0.976466	0.979813
GLRLMFeatures2Davg	Grey level variance	591.391	110.68
GLRLMFeatures2Davg	Run length variance	0.0257278	0.021467
GLRLMFeatures2Davg	Run entropy	5.65022	4.09108
GLRLMFeatures2DDmrg	short run emphasis	0.982153	0.983986
GLRLMFeatures2DDmrg	long runs emphasis	1.07741	1.06786
GLRLMFeatures2DDmrg	Low grey level run emphasis	0.0255311	0.059194
GLRLMFeatures2DDmrg	High grey level run emphasis	2446.26	557.77
GLRLMFeatures2DDmrg	Short run low grey level emphasis	0.0250214	0.058634
GLRLMFeatures2DDmrg	Short run high grey level emphasis	2383.49	545.847
GLRLMFeatures2DDmrg	Long run low grey level emphasis	0.0277585	0.061457
GLRLMFeatures2DDmrg	Long run high grey level emphasis	2717.29	607.536
GLRLMFeatures2DDmrg	Grey level non uniformity	71.7374	35.4012
GLRLMFeatures2DDmrg	Grey level non uniformity normalized	0.0112313	0.024136
GLRLMFeatures2DDmrg	Run length non uniformity	6093.79	1406.16
GLRLMFeatures2DDmrg	Run length non uniformity normalized	0.953998	0.958583
GLRLMFeatures2DDmrg	Run percentage	0.975749	0.978486
GLRLMFeatures2DDmrg	Grey level variance	785.802	175.835
GLRLMFeatures2DDmrg	Run length variance	0.0269803	0.023212
GLRLMFeatures2DDmrg	Run entropy	6.75124	5.6079
GLRLMFeatures2DWmrg	short run emphasis	0.98251	0.984799
GLRLMFeatures2DWmrg	long runs emphasis	1.07485	1.06385
GLRLMFeatures2DWmrg	Low grey level run emphasis	0.0324121	0.094445
GLRLMFeatures2DWmrg	High grey level run emphasis	2055.04	419.533
GLRLMFeatures2DWmrg	Short run low grey level emphasis	0.0317799	0.093787
GLRLMFeatures2DWmrg	Short run high grey level emphasis	2004.9	410.987
GLRLMFeatures2DWmrg	Long run low grey level emphasis	0.0350563	0.097095
GLRLMFeatures2DWmrg	Long run high grey level emphasis	2271.6	455.445
GLRLMFeatures2DWmrg	Grey level non uniformity	10.9891	7.60756
GLRLMFeatures2DWmrg	Grey level non uniformity normalized	0.0309069	0.102976
GLRLMFeatures2DWmrg	Run length non uniformity	554.013	144.253
GLRLMFeatures2DWmrg	Run length non uniformity normalized	0.954963	0.960947
GLRLMFeatures2DWmrg	Run percentage	0.976466	0.979813
GLRLMFeatures2DWmrg	Grey level variance	591.52	110.713
GLRLMFeatures2DWmrg	Run length variance	0.0257427	0.021532
GLRLMFeatures2DWmrg	Run entropy	5.71411	4.15618
GLRLMFeatures2Dvmrg	short run emphasis	0.982176	0.98403
GLRLMFeatures2Dvmrg	long runs emphasis	1.0773	1.06767
GLRLMFeatures2Dvmrg	Low grey level run emphasis	0.0255327	0.059191
GLRLMFeatures2Dvmrg	High grey level run emphasis	2446.26	557.776
GLRLMFeatures2Dvmrg	Short run low grey level emphasis	0.0250246	0.058631
GLRLMFeatures2Dvmrg	Short run high grey level emphasis	2383.55	545.883
GLRLMFeatures2Dvmrg	Long run low grey level emphasis	0.0277521	0.061453
GLRLMFeatures2Dvmrg	Long run high grey level emphasis	2717.04	607.414
GLRLMFeatures2Dvmrg	Grey level non uniformity	286.882	141.544
GLRLMFeatures2Dvmrg	Grey level non uniformity normalized	0.0112287	0.024126
GLRLMFeatures2Dvmrg	Run length non uniformity	24373.8	5624.04
GLRLMFeatures2Dvmrg	Run length non uniformity normalized	0.954002	0.958588
GLRLMFeatures2Dvmrg	Run percentage	0.975749	0.978486
GLRLMFeatures2Dvmrg	Grey level variance	2.01E+07	1.03E+06
GLRLMFeatures2Dvmrg	Run length variance	689.218	136.164
GLRLMFeatures2Dvmrg	Run entropy	-201228	-40420
GLRLMFeatures3Davg	short run emphasis	0.980412	0.981167
GLRLMFeatures3Davg	long runs emphasis	1.08557	1.08249
GLRLMFeatures3Davg	Low grey level run emphasis	0.0257903	0.05891
GLRLMFeatures3Davg	High grey level run emphasis	2444.81	558.033
GLRLMFeatures3Davg	Short run low grey level emphasis	0.0254143	0.057993
GLRLMFeatures3Davg	Short run high grey level emphasis	2377.03	544.775
GLRLMFeatures3Davg	Long run low grey level emphasis	0.0273925	0.062963
GLRLMFeatures3Davg	Long run high grey level emphasis	2741.96	615.531
GLRLMFeatures3Davg	Grey level non uniformity	71.5843	35.2249
GLRLMFeatures3Davg	Grey level non uniformity normalized	0.0112336	0.024114
GLRLMFeatures3Davg	Run length non uniformity	6053.15	1390.71
GLRLMFeatures3Davg	Run length non uniformity normalized	0.949712	0.951732
GLRLMFeatures3Davg	Run percentage	0.973478	0.974496
GLRLMFeatures3Davg	Grey level variance	786.11	175.765
GLRLMFeatures3Davg	Run length variance	0.0299645	0.028894
GLRLMFeatures3Davg	Run entropy	6.76268	5.62812
GLRLMFeatures3Dmrg	short run emphasis	0.980491	0.981291
GLRLMFeatures3Dmrg	long runs emphasis	1.08516	1.08189
GLRLMFeatures3Dmrg	Low grey level run emphasis	0.0257892	0.058913
GLRLMFeatures3Dmrg	High grey level run emphasis	2444.9	558.061
GLRLMFeatures3Dmrg	Short run low grey level emphasis	0.0254144	0.058007
GLRLMFeatures3Dmrg	Short run high grey level emphasis	2377.37	544.889
GLRLMFeatures3Dmrg	Long run low grey level emphasis	0.0273848	0.062922
GLRLMFeatures3Dmrg	Long run high grey level emphasis	2740.75	615.11
GLRLMFeatures3Dmrg	Grey level non uniformity	930.273	457.594
GLRLMFeatures3Dmrg	Grey level non uniformity normalized	0.0112296	0.024097
GLRLMFeatures3Dmrg	Run length non uniformity	78677.6	18074
GLRLMFeatures3Dmrg	Run length non uniformity normalized	0.949743	0.951766
GLRLMFeatures3Dmrg	Run percentage	0.973478	0.974496
GLRLMFeatures3Dmrg	Grey level variance	786.146	175.777
GLRLMFeatures3Dmrg	Run length variance	0.0299309	0.028857
GLRLMFeatures3Dmrg	Run entropy	6.78179	5.66008
GLSZMFeatures2Davg	small zone emphasis	0.933076	0.939815
GLSZMFeatures2Davg	Large zone emphasis	1.34218	1.29124
GLSZMFeatures2Davg	Low grey level zone emphasis	0.0320386	0.096903
GLSZMFeatures2Davg	High grey level zone emphasis	1995.37	410.732
GLSZMFeatures2Davg	Small zone low grey level emphasis	0.0294297	0.094436
GLSZMFeatures2Davg	Small zone high grey level emphasis	1818.57	376.276
GLSZMFeatures2Davg	Large zone low grey level emphasis	0.0438078	0.108737
GLSZMFeatures2Davg	Large zone high grey level emphasis	2997.25	574.297
GLSZMFeatures2Davg	Grey level non uniformity GLSZM	2.52354	1.7533
GLSZMFeatures2Davg	Grey level non uniformity normalized GLSZM	0.0302619	0.102261
GLSZMFeatures2Davg	Zone size non uniformity	114.018	30.1663
GLSZMFeatures2Davg	Zone size non uniformity normalized	0.842786	0.860976
GLSZMFeatures2Davg	Zone percentage GLSZM	0.909545	0.921198
GLSZMFeatures2Davg	Grey level variance GLSZM	577.607	110.069
GLSZMFeatures2Davg	Zone size variance	0.127283	0.100304
GLSZMFeatures2Davg	Zone size entropy	5.76471	4.16039
GLSZMFeatures2Dvmrg	small zone emphasis	0.934618	0.938805
GLSZMFeatures2Dvmrg	Large zone emphasis	1.35072	1.30153
GLSZMFeatures2Dvmrg	Low grey level zone emphasis	0.0251546	0.061019
GLSZMFeatures2Dvmrg	High grey level zone emphasis	2371.63	544.046
GLSZMFeatures2Dvmrg	Small zone low grey level emphasis	0.0230808	0.059012
GLSZMFeatures2Dvmrg	Small zone high grey level emphasis	2153.7	496.801
GLSZMFeatures2Dvmrg	Large zone low grey level emphasis	0.0350439	0.07091
GLSZMFeatures2Dvmrg	Large zone high grey level emphasis	3616.15	766.913
GLSZMFeatures2Dvmrg	Grey level non uniformity GLSZM	67.7991	33.6278
GLSZMFeatures2Dvmrg	Grey level non uniformity normalized GLSZM	0.0114101	0.024492
GLSZMFeatures2Dvmrg	Zone size non uniformity	5011.05	1170.12
GLSZMFeatures2Dvmrg	Zone size non uniformity normalized	0.843327	0.852234
GLSZMFeatures2Dvmrg	Zone percentage GLSZM	0.90773	0.915944
GLSZMFeatures2Dvmrg	Grey level variance GLSZM	767.831	172.677
GLSZMFeatures2Dvmrg	Zone size variance	0.137093	0.109568
GLSZMFeatures2Dvmrg	Zone size entropy	7.02124	5.86214
GLSZMFeatures3D	small zone emphasis	0.813499	0.79262
GLSZMFeatures3D	Large zone emphasis	3.12934	2.81191
GLSZMFeatures3D	Low grey level zone emphasis	0.0273345	0.061632
GLSZMFeatures3D	High grey level zone emphasis	2152.63	519.802
GLSZMFeatures3D	Small zone low grey level emphasis	0.022273	0.050623
GLSZMFeatures3D	Small zone high grey level emphasis	1620.14	389.07
GLSZMFeatures3D	Large zone low grey level emphasis	0.05964	0.154209
GLSZMFeatures3D	Large zone high grey level emphasis	9643.6	1750.46
GLSZMFeatures3D	Grey level non uniformity GLSZM	56.1058	26.4197
GLSZMFeatures3D	Grey level non uniformity normalized GLSZM	0.0120141	0.024971
GLSZMFeatures3D	Zone size non uniformity	2880.98	617.66
GLSZMFeatures3D	Zone size non uniformity normalized	0.616912	0.583799
GLSZMFeatures3D	Zone percentage GLSZM	0.713413	0.705804
GLSZMFeatures3D	Grey level variance GLSZM	715.552	165.878
GLSZMFeatures3D	Zone size variance	1.16454	0.804518
GLSZMFeatures3D	Zone size entropy	7.54889	6.50013
ngtdmFeatures2avg	coarseness	0.0471159	0.100679
ngtdmFeatures2avg	contrast	3.69375	3.76385
ngtdmFeatures2avg	busyness	0.0330006	0.090373
ngtdmFeatures2avg	complexity	19785.2	2763.6
ngtdmFeatures2avg	strength	140.446	40.8436
ngtdmFeatures2Dmrg	coarseness	0.00157834	0.003713
ngtdmFeatures2Dmrg	contrast	0.876573	0.967
ngtdmFeatures2Dmrg	busyness	0.218762	0.523067
ngtdmFeatures2Dmrg	complexity	55714.2	8060.35
ngtdmFeatures2Dmrg	strength	10.9094	4.23507
ngtdmFeatures3D	coarseness	0.00167336	0.004049
ngtdmFeatures3D	contrast	0.834316	0.892503
ngtdmFeatures3D	busyness	0.206339	0.47965
ngtdmFeatures3D	complexity	52577	7434.46
ngtdmFeatures3D	strength	11.4619	4.58857
gldzmFeatures2Davg	small distance emphasis GLDZM	0.485879	0.700448
gldzmFeatures2Davg	Large distance emphasis GLDZM	6.1895	2.67393
gldzmFeatures2Davg	Low grey level zone emphasis GLDZM	0.0320386	0.096903
gldzmFeatures2Davg	High grey level zone emphasis GLDZM	1995.37	410.732
gldzmFeatures2Davg	Small distance low grey level emphasis GLDZM	0.0315578	0.096197
gldzmFeatures2Davg	Small distance high grey level emphasis GLDZM	297.816	130.51
gldzmFeatures2Davg	Large distance low grey level emphasis GLDZM	0.0353462	0.100135
gldzmFeatures2Davg	Large distance high grey level emphasis GLDZM	24386.8	2261.63
gldzmFeatures2Davg	Grey level non uniformity GLDZM	2.52354	1.7533
gldzmFeatures2Davg	Grey level non uniformity normalized GLDZM	0.0302619	0.102261
gldzmFeatures2Davg	Zone distance non uniformity GLDZM	33.9535	15.1022
gldzmFeatures2Davg	Zone distance non uniformity normalized GLDZM	0.305874	0.51943
gldzmFeatures2Davg	Zone percentage GLDZM	0.909545	0.921198
gldzmFeatures2Davg	Grey level variance GLDZM	31.3469	0
gldzmFeatures2Davg	Zone distance variance GLDZM	1.29699	0.401122
gldzmFeatures2Davg	Zone distance entropy GLDZM	6.06286	4.23159
gldzmFeatures2Dmrg	small distance emphasis GLDZM	0.425335	0.629694
gldzmFeatures2Dmrg	Large distance emphasis GLDZM	7.27129	3.19519
gldzmFeatures2Dmrg	Low grey level zone emphasis GLDZM	0.0251546	0.061019
gldzmFeatures2Dmrg	High grey level zone emphasis GLDZM	2371.63	544.046
gldzmFeatures2Dmrg	Small distance low grey level emphasis GLDZM	0.0246857	0.060325
gldzmFeatures2Dmrg	Small distance high grey level emphasis GLDZM	324.83	158.264
gldzmFeatures2Dmrg	Large distance low grey level emphasis GLDZM	0.0287398	0.064406
gldzmFeatures2Dmrg	Large distance high grey level emphasis GLDZM	30951.9	3192.03
gldzmFeatures2Dmrg	Grey level non uniformity GLDZM	67.7991	33.6278
gldzmFeatures2Dmrg	Grey level non uniformity normalized GLDZM	0.0114101	0.024492
gldzmFeatures2Dmrg	Zone distance non uniformity GLDZM	1425.64	558.978
gldzmFeatures2Dmrg	Zone distance non uniformity normalized GLDZM	0.239925	0.407122
gldzmFeatures2Dmrg	Zone percentage GLDZM	0.226932	0.228986
gldzmFeatures2Dmrg	Grey level variance GLDZM	767.831	172.677
gldzmFeatures2Dmrg	Zone distance variance GLDZM	1.65642	0.569046
gldzmFeatures2Dmrg	Zone distance entropy GLDZM	7.91749	6.14384
gldzmFeatures3D	small distance emphasis GLDZM	0.469261	0.662652
gldzmFeatures3D	Large distance emphasis GLDZM	6.01905	2.9195
gldzmFeatures3D	Low grey level zone emphasis GLDZM	0.0271717	0.061783
gldzmFeatures3D	High grey level zone emphasis GLDZM	2170.54	524.408
gldzmFeatures3D	Small distance low grey level emphasis GLDZM	0.0268065	0.061231
gldzmFeatures3D	Small distance high grey level emphasis GLDZM	342.609	166.064
gldzmFeatures3D	Large distance low grey level emphasis GLDZM	0.0297729	0.064514
gldzmFeatures3D	Large distance high grey level emphasis GLDZM	24688	2833.94
gldzmFeatures3D	Grey level non uniformity GLDZM	52.7628	25.5975
gldzmFeatures3D	Grey level non uniformity normalized GLDZM	0.0119671	0.024828
gldzmFeatures3D	Zone distance non uniformity GLDZM	1199.29	449.805
gldzmFeatures3D	Zone distance non uniformity normalized GLDZM	0.27201	0.43628
gldzmFeatures3D	Zone percentage GLDZM	0.673541	0.687792
gldzmFeatures3D	Grey level variance GLDZM	723.5	167.231
gldzmFeatures3D	Zone distance variance GLDZM	1.35486	0.511129
gldzmFeatures3D	Zone distance entropy GLDZM	7.66533	6.0446
ngldmFeatures2Davg	Low dependence emphasis	0.871333	0.887265
ngldmFeatures2Davg	High dependence emphasis	1.61322	1.51255
ngldmFeatures2Davg	Low grey level count emphasis	0.0325137	0.093737
ngldmFeatures2Davg	High grey level count emphasis	2075.48	422.07
ngldmFeatures2Davg	Low dependence low grey level emphasis	0.0276252	0.089119
ngldmFeatures2Davg	Low dependence high grey level emphasis	1719.51	356.977
ngldmFeatures2Davg	High dependence low grey level emphasis	0.0533236	0.115751
ngldmFeatures2Davg	High dependence high grey level emphasis	3894.25	707.096
ngldmFeatures2Davg	Grey level non uniformity	2.85545	1.97342
ngldmFeatures2Davg	Grey level non uniformity normalized	0.0314145	0.103863
ngldmFeatures2Davg	Dependence count non uniformity	106.547	28.6239
ngldmFeatures2Davg	Dependence count non uniformity normalized	0.723421	0.764331
ngldmFeatures2Davg	Dependence count percentage	1	1
ngldmFeatures2Davg	Grey level variance	595.774	110.807
ngldmFeatures2Davg	Dependence count variance	0.194989	0.150106
ngldmFeatures2Davg	Dependence count entropy	5.8177	4.18319
ngldmFeatures2Davg	dependence Count Energy	0.0266979	0.098093
ngldmFeatures2Dmrg	Low dependence emphasis	0.870168	0.880351
ngldmFeatures2Dmrg	High dependence emphasis	1.64253	1.54837
ngldmFeatures2Dmrg	Low grey level count emphasis	0.025608	0.058652
ngldmFeatures2Dmrg	High grey level count emphasis	2472.08	561.663
ngldmFeatures2Dmrg	Low dependence low grey level emphasis	0.0216438	0.054694
ngldmFeatures2Dmrg	Low dependence high grey level emphasis	2028.56	470.824
ngldmFeatures2Dmrg	High dependence low grey level emphasis	0.0429454	0.077768
ngldmFeatures2Dmrg	High dependence high grey level emphasis	4760.04	957.841
ngldmFeatures2Dmrg	Grey level non uniformity	73.1607	36.038
ngldmFeatures2Dmrg	Grey level non uniformity normalized	0.0111764	0.024041
ngldmFeatures2Dmrg	Dependence count non uniformity	4665.79	1096.4
ngldmFeatures2Dmrg	Dependence count non uniformity normalized	0.712769	0.731422
ngldmFeatures2Dmrg	Dependence count percentage	1	1
ngldmFeatures2Dmrg	Grey level variance	791.594	176.755
ngldmFeatures2Dmrg	Dependence count variance	0.216866	0.174513
ngldmFeatures2Dmrg	Dependence count entropy	7.28585	6.09145
ngldmFeatures2Dmrg	dependence Count Energy	0.00834373	0.018491
ngldmFeatures3D	Low dependence emphasis	0.636942	0.621879
ngldmFeatures3D	High dependence emphasis	3.82004	3.48699
ngldmFeatures3D	Low grey level count emphasis	0.025608	0.058652
ngldmFeatures3D	High grey level count emphasis	2472.08	561.663
ngldmFeatures3D	Low dependence low grey level emphasis	0.017273	0.039185
ngldmFeatures3D	Low dependence high grey level emphasis	1326.35	313.408
ngldmFeatures3D	High dependence low grey level emphasis	0.0703404	0.176361
ngldmFeatures3D	High dependence high grey level emphasis	12867.8	2371.72
ngldmFeatures3D	Grey level non uniformity	73.1607	36.038
ngldmFeatures3D	Grey level non uniformity normalized	0.0111764	0.024041
ngldmFeatures3D	Dependence count non uniformity	2626.57	598.625
ngldmFeatures3D	Dependence count non uniformity normalized	0.401248	0.39935
ngldmFeatures3D	Dependence count percentage	1	1
ngldmFeatures3D	Grey level variance	791.594	176.755
ngldmFeatures3D	Dependence count variance	0.965357	0.721061
ngldmFeatures3D	Dependence count entropy	8.06942	6.88305
ngldmFeatures3D	dependence Count Energy	0.00500475	0.010548

## Discussion

We developed a radiomics calculator that is easy to use and can be called from any programming language. It includes the most frequently used preprocessing steps and complies with the IBSI standards. It can handle several input image formats as well as different VOI types. The created output files are organized in a way that eases further processing of the feature values. In this way, the calculator can be included easily in any radiomics pipeline and the results can be used for further analysis. Furthermore, all preprocessing steps are reported, so that a valid documentation of the performed preprocessing steps can easily be extracted from the output files.

To make radiomic studies comparable across studies and institutions, it is essential that the different radiomic software packages calculate the same feature values for every defined feature. Therefore, the standardization of feature definitions and calculations is essential [19,20]. IBSI provides benchmark feature definitions and feature values extracted from phantom scans. As RaCaT follows these definitions and calculates feature values in compliance with these standard, it could be used to standardize other software packages.

Some small deviations were found in the calculation of the morphological features when compared with the IBSI standard. These deviations include the calculated volume and surface of the object and therefore all features depending on these two values. These deviations are due to a different implementation of the 3D presentation of the image mask. Also when comparing the morphological features extracted from the spheres of the NEMA image quality phantom, the deviations between ideal and calculated volume were in the majority of the cases small. Only for the smaller spheres, the deviation increased. This increase in deviation is likely more due to the partial volume effect than to mistakes in the implementation. The partial volume effect has especially an impact on smaller objects.

One limitation of RaCaT is that it does not provide any Graphical User Interface or automatic algorithm to perform segmentation tasks. It calculates radiomic features from previous performed segmentations. Moreover, after feature calculation, it provides also no further processing of the calculated features. I.e. no machine or deep learning algorithm are implemented and RaCaT can therefore not directly be used to build predictive models. However, as it can be called by any programming language, it can easily be included in any machine or deep learning script.

## Further development

The following additional features will be implemented in further releases:

### Additional discretization methods

Many other ways for image discretization have been proposed. Among them intensity histogram equalization and the Lloyd-max algorithm [21]. The next release will include both discretization methods.

### Read several DICOM series stored in one folder

When images are extracted from the scanner, different image series are often stored in one folder. For a future release, it will be possible to read a folder containing several DICOM image series and the program will calculate for every DICOM image series the features separately.

### Several tumors in one mask

Up to now, the calculator can only handle masks that come with one marked VOI. For future releases, it will be possible that more than one VOI can be marked in one mask and the feature values of the different VOIs will be calculated separately.

### Additional distances for the calculation of textural matrices

In the current version of RaCaT only distance 1 is used for the calculation of textural matrices as this is the common distance for calculations. In future releases, also other distances can be set by the user.

### Additional output formats

Up to now, the output is only available as .csv file. It is planned that an output in ontology format [22] is also available.

## Conclusion

We implemented and tested successfully RaCaT, an easy to use Radiomics calculator that can be included in any programming language or used from the command line. The calculated features are meeting the IBSI standards. The calculator is ready to use without requiring any programming skills, but can also be downloaded, built from source and extended if needed. As the implementation of the calculator is highly modularized, it is easily extendable. A documentation including the description of how to use the calculator as well as a more extensive description of the programming concepts, can be found on GitHub.

## Supporting information

S1 Fig Example commands to call executable. (DOCX)

S1 Table Feature values for mathematical phantom provided by the image biomarker standardization initiative. Benchmark feature values and values calculated by RaCaT as well as their differences and percentage differences for the mathematical digital phantom provided by the Image Biomarker standardization initiative.
(DOCX)

S2 Table Feature values for realistic phantom provided by IBSI–config A. Benchmark feature values and values calculated by RaCaT as well as their differences and percentage differences for the realistic phantom, config A provided by IBSI.
(DOCX)

S3 Table Feature values for realistic phantom provided by IBSI–config C. Benchmark feature values and values calculated by RaCaT as well as their differences and percentage differences for the realistic phantom, config C provided by IBSI.
(DOCX)
