# Supplementary material for: RaCaT: An open source and easy to use radiomics calculator tool
Source: PLoS One. 2019 Feb 20;14(2):e0212223. doi: 10.1371/journal.pone.0212223 (PMC6382170; doi:10.1371/journal.pone.0212223)
Supplement: S1 Fig — (DOCX) [file pone.0212223.s001.docx]

| Call executable for nifti image and mask | C:/ExampleData_Radiomics/Racat_v11.exe --ini C:/ExampleData_Radiomics/config_PET_FBN_NoInterpolation.ini --out C:/ExampleData_Radiomics/features_PET_FBN_NoInterpolation --voi C:/ExampleData_Radiomics/sphere1.nii --img C:/ExampleData_Radiomics/nemaScan.nii --pat C:/ExampleData_Radiomics/patientInfo.ini |
| --- | --- |
| Call executable for nifti image and mask and feature output definition file | C:/ExampleData_Radiomics/Racat_v11.exe --ini C:/ExampleData_Radiomics/config_PET_FBN_NoInterpolation.ini --out C:/ExampleData_Radiomics/features_PET_FBN_NoInterpolation3D --voi C:/ExampleData_Radiomics/sphere1.nii --img C:/ExampleData_Radiomics/nemaScan.nii --pat C:/ExampleData_Radiomics/patientInfo.ini --fts C:/ExampleData_Radiomics/featureDefinition_3D.ini |
| Call executable for DICOM image and mask | C:/ExampleData_Radiomics/Racat_v11.exe --ini C:/ExampleData_Radiomics/config_PET_FBW_NoInterpolation.ini --out C:/ExampleData_Radiomics/features_PET_FBW_NoInterpolation_dicom --voi C:/ExampleData_Radiomics/sphere1.nii --img C:/ExampleData_Radiomics/Dicom_NEMA --pat C:/ExampleData_Radiomics/patientInfo.ini |
| Call executable for nifti image and rt-struct mask | C:/ExampleData_Radiomics/Racat_v11.exe --ini C:/ExampleData_Radiomics/config_PET_FBW_NoInterpolation.ini --out C:/ExampleData_Radiomics/features_PET_FBW_NoInterpolation_DCM_RTS --rts C:/ExampleData_Radiomics/sphere1_RS.dcm --img C:/ExampleData_Radiomics/Dicom_NEMA --pat C:/ExampleData_Radiomics/patientInfo.ini |

S1 Fig: Example commands to call executable
