# Supplementary material for: RaCaT: An open source and easy to use radiomics calculator tool
Source: PLoS One. 2019 Feb 20;14(2):e0212223. doi: 10.1371/journal.pone.0212223 (PMC6382170; doi:10.1371/journal.pone.0212223)
Supplement: S1 Table — Benchmark feature values and values calculated by RaCaT as well as their differences and percentage differences for the mathematical digital phantom provided by the Image Biomarker standardization initiative. (DOCX) [file pone.0212223.s002.docx]

S1 Table: Benchmark feature values and values calculated by RaCaT as well as their differences and percentage differences for the mathematical digital phantom provided by the Image Biomarker standardization initiative

| **data_set** | **family** | **image_biomarker** | **benchmark_value** | **RaCaT** | **difference** | **%difference** |
| --- | --- | --- | --- | --- | --- | --- |
| digital phantom | Morphology | Volume (mesh-based) | 556 | 592 | 36 | 6,47482 |
| digital phantom | Morphology | Volume (counting) | 592 | 592 | 0 | 0 |
| digital phantom | Morphology | Surface area | 388 | 370,043 | 17 | 4,381443 |
| digital phantom | Morphology | Surface to volume ratio | 0,698 | 0,625073 | 0,072 | 10,31519 |
| digital phantom | Morphology | Compactness 1 | 0,0411 | 0,046921 | 0,0058 | 14,11192 |
| digital phantom | Morphology | Compactness 2 | 0,599 | 0,782238 | 0,183 | 30,55092 |
| digital phantom | Morphology | Spherical disproportion | 1,19 | 1,08531 | 0,1 | 8,403361 |
| digital phantom | Morphology | Sphericity | 0,843 | 0,921396 | 0,078 | 9,252669 |
| digital phantom | Morphology | Asphericity | 0,186 | 0,085309 | 0,1 | 53,76344 |
| digital phantom | Morphology | Centre of mass shift | 0,672 | 0,671545 | 0 | 0 |
| digital phantom | Morphology | Maximum 3D diameter | 11,7 | 11,6619 | 0 | 0 |
| digital phantom | Morphology | Major axis length | 11,4 | 11,3251 | 0 | 0 |
| digital phantom | Morphology | Minor axis length | 9,31 | 9,2449 | 0,06 | 0,644468 |
| digital phantom | Morphology | Least axis length | 8,54 | 8,47811 | 0,06 | 0,702576 |
| digital phantom | Morphology | Elongation | 0,816 | 0,903505 | 0,087 | 10,66176 |
| digital phantom | Morphology | Flatness | 0,749 | 0,865225 | 0,116 | 15,48732 |
| digital phantom | Morphology | Volume density (AABB) | 0,869 | 0,925 | 0,056 | 6,444189 |
| digital phantom | Morphology | Area density (AABB) | 0,866 | 0,825989 | 0,04 | 4,618938 |
| digital phantom | Morphology | Volume density (AEE) | 1,17 | 1,27374 | 0,1 | 8,547009 |
| digital phantom | Morphology | Integrated intensity | 1270 | 1272 | 0 | 0 |
| digital phantom | Morphology | Moran's I index | 0,0397 | 0,039704 | 0 | 0 |
| digital phantom | Morphology | Geary's C measure | 0,974 | 0,974036 | 0 | 0 |
| digital phantom | Local intensity | Local intensity peak | 2,6 | 2,5993 | 0 | 0 |
| digital phantom | Local intensity | Global intensity peak | 3,1 | 3,41463 | 0,31 | 10 |
| digital phantom | Statistics | Mean | 2,15 | 2,14865 | 0 | 0 |
| digital phantom | Statistics | Variance | 3,05 | 3,04547 | 0 | 0 |
| digital phantom | Statistics | Skewness | 1,08 | 1,08382 | 0 | 0 |
| digital phantom | Statistics | (Excess) kurtosis | -0,355 | -0,35462 | 0 | 0 |
| digital phantom | Statistics | Median | 1 | 1 | 0 | 0 |
| digital phantom | Statistics | Minimum | 1 | 1 | 0 | 0 |
| digital phantom | Statistics | 10th percentile | 1 | 1 | 0 | 0 |
| digital phantom | Statistics | 90th percentile | 4 | 4 | 0 | 0 |
| digital phantom | Statistics | Maximum | 6 | 6 | 0 | 0 |
| digital phantom | Statistics | Interquartile range | 3 | 3 | 0 | 0 |
| digital phantom | Statistics | Range | 5 | 5 | 0 | 0 |
| digital phantom | Statistics | Mean absolute deviation | 1,55 | 1,55223 | 0 | 0 |
| digital phantom | Statistics | Robust mean absolute deviation | 1,11 | 1,11383 | 0 | 0 |
| digital phantom | Statistics | Median absolute deviation | 1,15 | 1,14865 | 0 | 0 |
| digital phantom | Statistics | Coefficient of variation | 0,812 | 0,812198 | 0 | 0 |
| digital phantom | Statistics | Quartile coefficient of dispersion | 0,6 | 0,6 | 0 | 0 |
| digital phantom | Statistics | Energy | 567 | 567 | 0 | 0 |
| digital phantom | Statistics | Root mean square | 2,77 | 2,76806 | 0 | 0 |
| digital phantom | Intensity histogram | Mean | 2,15 | 2,14865 | 0 | 0 |
| digital phantom | Intensity histogram | Variance | 3,05 | 3,04547 | 0 | 0 |
| digital phantom | Intensity histogram | Skewness | 1,08 | 1,08382 | 0 | 0 |
| digital phantom | Intensity histogram | Kurtosis | -0,355 | -0,35462 | 0 | 0 |
| digital phantom | Intensity histogram | Median | 1 | 1 | 0 | 0 |
| digital phantom | Intensity histogram | Minimum | 1 | 1 | 0 | 0 |
| digital phantom | Intensity histogram | 10th percentile | 1 | 1 | 0 | 0 |
| digital phantom | Intensity histogram | 90th percentile | 4 | 4 | 0 | 0 |
| digital phantom | Intensity histogram | Maximum | 6 | 6 | 0 | 0 |
| digital phantom | Intensity histogram | Mode | 1 | 1 | 0 | 0 |
| digital phantom | Intensity histogram | Interquartile range | 3 | 3 | 0 | 0 |
| digital phantom | Intensity histogram | Range | 5 | 5 | 0 | 0 |
| digital phantom | Intensity histogram | Mean absolute deviation | 1,55 | 1,55223 | 0 | 0 |
| digital phantom | Intensity histogram | Robust mean absolute deviation | 1,11 | 1,11383 | 0 | 0 |
| digital phantom | Intensity histogram | Median absolute deviation | 1,15 | 1,14865 | 0 | 0 |
| digital phantom | Intensity histogram | Coefficient of variation | 0,812 | 0,812198 | 0 | 0 |
| digital phantom | Intensity histogram | Quartile coefficient of dispersion | 0,6 | 0,6 | 0 | 0 |
| digital phantom | Intensity histogram | Entropy | 1,27 | 1,26561 | 0 | 0 |
| digital phantom | Intensity histogram | Uniformity | 0,512 | 0,512418 | 0 | 0 |
| digital phantom | Intensity histogram | Maximum histogram gradient | 8 | 8 | 0 | 0 |
| digital phantom | Intensity histogram | Maximum gradient grey level | 3 | 4 | 1 | 33,33333 |
| digital phantom | Intensity histogram | Minimum histogram gradient | -50 | -50 | 0 | 0 |
| digital phantom | Intensity histogram | Minimum gradient grey level | 1 | 2 | 1 | 100 |
| digital phantom | Intensity volume histogram | Volume fraction at 10% intensity | 0,324 | 0,324324 | 0 | 0 |
| digital phantom | Intensity volume histogram | Volume fraction at 90% intensity | 0,0946 | 0,094595 | 0 | 0 |
| digital phantom | Intensity volume histogram | Intensity at 10% volume | 5 | 5 | 0 | 0 |
| digital phantom | Intensity volume histogram | Intensity at 90% volume | 2 | 2 | 0 | 0 |
| digital phantom | Intensity volume histogram | Volume fraction difference between 10% and 90% intensity | 0,23 | 0,22973 | 0 | 0 |
| digital phantom | Intensity volume histogram | Intensity difference between 10% and 90% volume | 3 | 3 | 0 | 0 |
| digital phantom | Intensity volume histogram | Area under the IVH curve | 0,32 |  |  | ######## |
| digital phantom | Co-occurrence matrix (2D, averaged) | Joint maximum | 0,519 | 0,5188 | 0 | 0 |
| digital phantom | Co-occurrence matrix (2D, averaged) | Joint average | 2,14 | 2,14242 | 0 | 0 |
| digital phantom | Co-occurrence matrix (2D, averaged) | Joint variance | 2,69 | 2,6877 | 0 | 0 |
| digital phantom | Co-occurrence matrix (2D, averaged) | Joint entropy | 2,05 | 2,04966 | 0 | 0 |
| digital phantom | Co-occurrence matrix (2D, averaged) | Difference average | 1,42 | 1,42247 | 0 | 0 |
| digital phantom | Co-occurrence matrix (2D, averaged) | Difference variance | 2,9 | 2,90159 | 0 | 0 |
| digital phantom | Co-occurrence matrix (2D, averaged) | Difference entropy | 1,4 | 1,39615 | 0 | 0 |
| digital phantom | Co-occurrence matrix (2D, averaged) | Sum average | 4,28 | 4,28484 | 0 | 0 |
| digital phantom | Co-occurrence matrix (2D, averaged) | Sum variance | 5,47 | 5,47293 | 0 | 0 |
| digital phantom | Co-occurrence matrix (2D, averaged) | Sum entropy | 1,6 | 1,60319 | 0 | 0 |
| digital phantom | Co-occurrence matrix (2D, averaged) | Angular second moment | 0,368 | 0,367529 | 0 | 0 |
| digital phantom | Co-occurrence matrix (2D, averaged) | Contrast | 5,28 | 5,27785 | 0 | 0 |
| digital phantom | Co-occurrence matrix (2D, averaged) | Dissimilarity | 1,42 | 1,42247 | 0 | 0 |
| digital phantom | Co-occurrence matrix (2D, averaged) | Inverse difference | 0,678 | 0,677949 | 0 | 0 |
| digital phantom | Co-occurrence matrix (2D, averaged) | Inverse difference normalised | 0,851 | 0,851399 | 0 | 0 |
| digital phantom | Co-occurrence matrix (2D, averaged) | Inverse difference moment | 0,619 | 0,618737 | 0 | 0 |
| digital phantom | Co-occurrence matrix (2D, averaged) | Inverse difference moment normalised | 0,899 | 0,899219 | 0 | 0 |
| digital phantom | Co-occurrence matrix (2D, averaged) | Inverse variance | 0,0567 | 0,056698 | 0 | 0 |
| digital phantom | Co-occurrence matrix (2D, averaged) | Correlation | -0,0121 | -0,01211 | 0 | 0 |
| digital phantom | Co-occurrence matrix (2D, averaged) | Autocorrelation | 5,09 | 5,09437 | 0 | 0 |
| digital phantom | Co-occurrence matrix (2D, averaged) | Cluster tendency | 5,47 | 5,47293 | 0 | 0 |
| digital phantom | Co-occurrence matrix (2D, averaged) | Cluster shade | 7 | 6,99782 | 0 | 0 |
| digital phantom | Co-occurrence matrix (2D, averaged) | Cluster prominence | 79,1 | 79,1126 | 0 | 0 |
| digital phantom | Co-occurrence matrix (2D, averaged) | Information correlation 1 | -0,155 | -0,15512 | 0 | 0 |
| digital phantom | Co-occurrence matrix (2D, averaged) | Information correlation 2 | 0,487 | 0,487457 | 0 | 0 |
| digital phantom | Co-occurrence matrix (2D, slice-merged) | Joint maximum | 0,512 | 0,512292 | 0 | 0 |
| digital phantom | Co-occurrence matrix (2D, slice-merged) | Joint average | 2,14 | 2,14343 | 0 | 0 |
| digital phantom | Co-occurrence matrix (2D, slice-merged) | Joint variance | 2,71 | 2,71158 | 0 | 0 |
| digital phantom | Co-occurrence matrix (2D, slice-merged) | Joint entropy | 2,24 | 2,23838 | 0 | 0 |
| digital phantom | Co-occurrence matrix (2D, slice-merged) | Difference average | 1,4 | 1,39902 | 0 | 0 |
| digital phantom | Co-occurrence matrix (2D, slice-merged) | Difference variance | 3,06 | 3,06426 | 0 | 0 |
| digital phantom | Co-occurrence matrix (2D, slice-merged) | Difference entropy | 1,49 | 1,49262 | 0 | 0 |
| digital phantom | Co-occurrence matrix (2D, slice-merged) | Sum average | 4,29 | 4,28686 | 0 | 0 |
| digital phantom | Co-occurrence matrix (2D, slice-merged) | Sum variance | 5,66 | 5,65615 | 0 | 0 |
| digital phantom | Co-occurrence matrix (2D, slice-merged) | Sum entropy | 1,79 | 1,79494 | 0 | 0 |
| digital phantom | Co-occurrence matrix (2D, slice-merged) | Angular second moment | 0,352 | 0,351678 | 0 | 0 |
| digital phantom | Co-occurrence matrix (2D, slice-merged) | Contrast | 5,19 | 5,19019 | 0 | 0 |
| digital phantom | Co-occurrence matrix (2D, slice-merged) | Dissimilarity | 1,4 | 1,39902 | 0 | 0 |
| digital phantom | Co-occurrence matrix (2D, slice-merged) | Inverse difference | 0,683 | 0,683294 | 0 | 0 |
| digital phantom | Co-occurrence matrix (2D, slice-merged) | Inverse difference normalised | 0,854 | 0,853846 | 0 | 0 |
| digital phantom | Co-occurrence matrix (2D, slice-merged) | Inverse difference moment | 0,625 | 0,625003 | 0 | 0 |
| digital phantom | Co-occurrence matrix (2D, slice-merged) | Inverse difference moment normalised | 0,901 | 0,900879 | 0 | 0 |
| digital phantom | Co-occurrence matrix (2D, slice-merged) | Inverse variance | 0,0553 | 0,055286 | 0 | 0 |
| digital phantom | Co-occurrence matrix (2D, slice-merged) | Correlation | 0,0173 | 0,017307 | 0 | 0 |
| digital phantom | Co-occurrence matrix (2D, slice-merged) | Autocorrelation | 5,14 | 5,13953 | 0 | 0 |
| digital phantom | Co-occurrence matrix (2D, slice-merged) | Cluster tendency | 5,66 | 5,65615 | 0 | 0 |
| digital phantom | Co-occurrence matrix (2D, slice-merged) | Cluster shade | 6,98 | 6,97661 | 0 | 0 |
| digital phantom | Co-occurrence matrix (2D, slice-merged) | Cluster prominence | 80,4 | 80,3855 | 0 | 0 |
| digital phantom | Co-occurrence matrix (2D, slice-merged) | Information correlation 1 | -0,0341 | -0,03409 | 0 | 0 |
| digital phantom | Co-occurrence matrix (2D, slice-merged) | Information correlation 2 | 0,263 | 0,262509 | 0 | 0 |
| digital phantom | Co-occurrence matrix (3D, averaged) | Joint maximum | 0,503 | 0,502811 | 0 | 0 |
| digital phantom | Co-occurrence matrix (3D, averaged) | Joint average | 2,14 | 2,143 | 0 | 0 |
| digital phantom | Co-occurrence matrix (3D, averaged) | Joint variance | 3,1 | 3,09932 | 0 | 0 |
| digital phantom | Co-occurrence matrix (3D, averaged) | Joint entropy | 2,4 | 2,39971 | 0 | 0 |
| digital phantom | Co-occurrence matrix (3D, averaged) | Difference average | 1,43 | 1,43098 | 0 | 0 |
| digital phantom | Co-occurrence matrix (3D, averaged) | Difference variance | 3,06 | 3,05628 | 0 | 0 |
| digital phantom | Co-occurrence matrix (3D, averaged) | Difference entropy | 1,56 | 1,56273 | 0 | 0 |
| digital phantom | Co-occurrence matrix (3D, averaged) | Sum average | 4,29 | 4,28599 | 0 | 0 |
| digital phantom | Co-occurrence matrix (3D, averaged) | Sum variance | 7,07 | 7,0728 | 0 | 0 |
| digital phantom | Co-occurrence matrix (3D, averaged) | Sum entropy | 1,92 | 1,92261 | 0 | 0 |
| digital phantom | Co-occurrence matrix (3D, averaged) | Angular second moment | 0,303 | 0,302975 | 0 | 0 |
| digital phantom | Co-occurrence matrix (3D, averaged) | Contrast | 5,32 | 5,32448 | 0 | 0 |
| digital phantom | Co-occurrence matrix (3D, averaged) | Dissimilarity | 1,43 | 1,43098 | 0 | 0 |
| digital phantom | Co-occurrence matrix (3D, averaged) | Inverse difference | 0,677 | 0,676615 | 0 | 0 |
| digital phantom | Co-occurrence matrix (3D, averaged) | Inverse difference normalised | 0,851 | 0,850679 | 0 | 0 |
| digital phantom | Co-occurrence matrix (3D, averaged) | Inverse difference moment | 0,618 | 0,617739 | 0 | 0 |
| digital phantom | Co-occurrence matrix (3D, averaged) | Inverse difference moment normalised | 0,898 | 0,898443 | 0 | 0 |
| digital phantom | Co-occurrence matrix (3D, averaged) | Inverse variance | 0,0604 | 0,060416 | 0 | 0 |
| digital phantom | Co-occurrence matrix (3D, averaged) | Correlation | 0,157 | 0,15735 | 0 | 0 |
| digital phantom | Co-occurrence matrix (3D, averaged) | Autocorrelation | 5,06 | 5,05544 | 0 | 0 |
| digital phantom | Co-occurrence matrix (3D, averaged) | Cluster tendency | 7,07 | 7,0728 | 0 | 0 |
| digital phantom | Co-occurrence matrix (3D, averaged) | Cluster shade | 16,6 | 16,6441 | 0 | 0 |
| digital phantom | Co-occurrence matrix (3D, averaged) | Cluster prominence | 145 | 144,703 | 0 | 0 |
| digital phantom | Co-occurrence matrix (3D, averaged) | Information correlation 1 | -0,157 | -0,15685 | 0 | 0 |
| digital phantom | Co-occurrence matrix (3D, averaged) | Information correlation 2 | 0,52 | 0,519588 | 0 | 0 |
| digital phantom | Co-occurrence matrix (3D, merged) | Joint maximum | 0,509 | 0,508539 | 0 | 0 |
| digital phantom | Co-occurrence matrix (3D, merged) | Joint average | 2,15 | 2,14896 | 0 | 0 |
| digital phantom | Co-occurrence matrix (3D, merged) | Joint variance | 3,13 | 3,13246 | 0 | 0 |
| digital phantom | Co-occurrence matrix (3D, merged) | Joint entropy | 2,57 | 2,5739 | 0 | 0 |
| digital phantom | Co-occurrence matrix (3D, merged) | Difference average | 1,38 | 1,37951 | 0 | 0 |
| digital phantom | Co-occurrence matrix (3D, merged) | Difference variance | 3,21 | 3,21461 | 0 | 0 |
| digital phantom | Co-occurrence matrix (3D, merged) | Difference entropy | 1,64 | 1,64088 | 0 | 0 |
| digital phantom | Co-occurrence matrix (3D, merged) | Sum average | 4,3 | 4,29791 | 0 | 0 |
| digital phantom | Co-occurrence matrix (3D, merged) | Sum variance | 7,41 | 7,4122 | 0 | 0 |
| digital phantom | Co-occurrence matrix (3D, merged) | Sum entropy | 2,11 | 2,10986 | 0 | 0 |
| digital phantom | Co-occurrence matrix (3D, merged) | Angular second moment | 0,291 | 0,290951 | 0 | 0 |
| digital phantom | Co-occurrence matrix (3D, merged) | Contrast | 5,12 | 5,11765 | 0 | 0 |
| digital phantom | Co-occurrence matrix (3D, merged) | Dissimilarity | 1,38 | 1,37951 | 0 | 0 |
| digital phantom | Co-occurrence matrix (3D, merged) | Inverse difference | 0,688 | 0,687698 | 0 | 0 |
| digital phantom | Co-occurrence matrix (3D, merged) | Inverse difference normalised | 0,856 | 0,855898 | 0 | 0 |
| digital phantom | Co-occurrence matrix (3D, merged) | Inverse difference moment | 0,631 | 0,630638 | 0 | 0 |
| digital phantom | Co-occurrence matrix (3D, merged) | Inverse difference moment normalised | 0,902 | 0,90221 | 0 | 0 |
| digital phantom | Co-occurrence matrix (3D, merged) | Inverse variance | 0,0574 | 0,057445 | 0 | 0 |
| digital phantom | Co-occurrence matrix (3D, merged) | Correlation | 0,183 | 0,183127 | 0 | 0 |
| digital phantom | Co-occurrence matrix (3D, merged) | Autocorrelation | 5,19 | 5,19165 | 0 | 0 |
| digital phantom | Co-occurrence matrix (3D, merged) | Cluster tendency | 7,41 | 7,4122 | 0 | 0 |
| digital phantom | Co-occurrence matrix (3D, merged) | Cluster shade | 17,4 | 17,4192 | 0 | 0 |
| digital phantom | Co-occurrence matrix (3D, merged) | Cluster prominence | 147 | 147,464 | 0 | 0 |
| digital phantom | Co-occurrence matrix (3D, merged) | Information correlation 1 | -0,0288 | -0,0288 | 0 | 0 |
| digital phantom | Co-occurrence matrix (3D, merged) | Information correlation 2 | 0,269 | 0,26917 | 0 | 0 |
| digital phantom | Run length matrix (2D, averaged) | Short runs emphasis | 0,641 | 0,640624 | 0 | 0 |
| digital phantom | Run length matrix (2D, averaged) | Long runs emphasis | 3,78 | 3,77838 | 0 | 0 |
| digital phantom | Run length matrix (2D, averaged) | Low grey level run emphasis | 0,604 | 0,604358 | 0 | 0 |
| digital phantom | Run length matrix (2D, averaged) | High grey level run emphasis | 9,82 | 9,82427 | 0 | 0 |
| digital phantom | Run length matrix (2D, averaged) | Short run low grey level emphasis | 0,294 | 0,293966 | 0 | 0 |
| digital phantom | Run length matrix (2D, averaged) | Short run high grey level emphasis | 8,57 | 8,57314 | 0 | 0 |
| digital phantom | Run length matrix (2D, averaged) | Long run low grey level emphasis | 3,14 | 3,14448 | 0 | 0 |
| digital phantom | Run length matrix (2D, averaged) | Long run high grey level emphasis | 17,4 | 17,387 | 0 | 0 |
| digital phantom | Run length matrix (2D, averaged) | Grey level non-uniformity | 5,2 | 5,19706 | 0 | 0 |
| digital phantom | Run length matrix (2D, averaged) | Grey level non-uniformity normalised | 0,46 | 0,459729 | 0 | 0 |
| digital phantom | Run length matrix (2D, averaged) | Run length non-uniformity | 6,12 | 6,12286 | 0 | 0 |
| digital phantom | Run length matrix (2D, averaged) | Run length non-uniformity normalised | 0,492 | 0,491741 | 0 | 0 |
| digital phantom | Run length matrix (2D, averaged) | Run percentage | 0,627 | 0,627099 | 0 | 0 |
| digital phantom | Run length matrix (2D, averaged) | Grey level variance | 3,35 | 3,35303 | 0 | 0 |
| digital phantom | Run length matrix (2D, averaged) | Run length variance | 0,761 | 0,761475 | 0 | 0 |
| digital phantom | Run length matrix (2D, averaged) | Run entropy | 2,17 | 2,16955 | 0 | 0 |
| digital phantom | Run length matrix (2D, slice-merged) | Short runs emphasis | 0,661 | 0,661197 | 0 | 0 |
| digital phantom | Run length matrix (2D, slice-merged) | Long runs emphasis | 3,51 | 3,51191 | 0 | 0 |
| digital phantom | Run length matrix (2D, slice-merged) | Low grey level run emphasis | 0,609 | 0,608521 | 0 | 0 |
| digital phantom | Run length matrix (2D, slice-merged) | High grey level run emphasis | 9,74 | 9,74264 | 0 | 0 |
| digital phantom | Run length matrix (2D, slice-merged) | Short run low grey level emphasis | 0,311 | 0,31081 | 0 | 0 |
| digital phantom | Run length matrix (2D, slice-merged) | Short run high grey level emphasis | 8,67 | 8,67312 | 0 | 0 |
| digital phantom | Run length matrix (2D, slice-merged) | Long run low grey level emphasis | 2,92 | 2,9201 | 0 | 0 |
| digital phantom | Run length matrix (2D, slice-merged) | Long run high grey level emphasis | 16,1 | 16,119 | 0 | 0 |
| digital phantom | Run length matrix (2D, slice-merged) | Grey level non-uniformity | 20,5 | 20,4873 | 0 | 0 |
| digital phantom | Run length matrix (2D, slice-merged) | Grey level non-uniformity normalised | 0,456 | 0,455529 | 0 | 0 |
| digital phantom | Run length matrix (2D, slice-merged) | Run length non-uniformity | 21,6 | 21,5992 | 0 | 0 |
| digital phantom | Run length matrix (2D, slice-merged) | Run length non-uniformity normalised | 0,441 | 0,441097 | 0 | 0 |
| digital phantom | Run length matrix (2D, slice-merged) | Run percentage | 0,627 | 0,627099 | 0 | 0 |
| digital phantom | Run length matrix (2D, slice-merged) | Grey level variance | 3,37 | 3,37419 | 0 | 0 |
| digital phantom | Run length matrix (2D, slice-merged) | Run length variance | 0,778 | 0,778184 | 0 | 0 |
| digital phantom | Run length matrix (2D, slice-merged) | Run entropy | 2,57 | 2,57011 | 0 | 0 |
| digital phantom | Run length matrix (3D, averaged) | Short runs emphasis | 0,705 | 0,705235 | 0 | 0 |
| digital phantom | Run length matrix (3D, averaged) | Long runs emphasis | 3,06 | 3,06112 | 0 | 0 |
| digital phantom | Run length matrix (3D, averaged) | Low grey level run emphasis | 0,603 | 0,60298 | 0 | 0 |
| digital phantom | Run length matrix (3D, averaged) | High grey level run emphasis | 9,7 | 9,69762 | 0 | 0 |
| digital phantom | Run length matrix (3D, averaged) | Short run low grey level emphasis | 0,352 | 0,35158 | 0 | 0 |
| digital phantom | Run length matrix (3D, averaged) | Short run high grey level emphasis | 8,54 | 8,53966 | 0 | 0 |
| digital phantom | Run length matrix (3D, averaged) | Long run low grey level emphasis | 2,39 | 2,39097 | 0 | 0 |
| digital phantom | Run length matrix (3D, averaged) | Long run high grey level emphasis | 17,6 | 17,5662 | 0 | 0 |
| digital phantom | Run length matrix (3D, averaged) | Grey level non-uniformity | 21,8 | 21,7762 | 0 | 0 |
| digital phantom | Run length matrix (3D, averaged) | Grey level non-uniformity normalised | 0,43 | 0,430175 | 0 | 0 |
| digital phantom | Run length matrix (3D, averaged) | Run length non-uniformity | 26,9 | 26,8534 | 0 | 0 |
| digital phantom | Run length matrix (3D, averaged) | Run length non-uniformity normalised | 0,513 | 0,512771 | 0 | 0 |
| digital phantom | Run length matrix (3D, averaged) | Run percentage | 0,68 | 0,679834 | 0 | 0 |
| digital phantom | Run length matrix (3D, averaged) | Grey level variance | 3,46 | 3,46498 | 0 | 0 |
| digital phantom | Run length matrix (3D, averaged) | Run length variance | 0,574 | 0,573542 | 0 | 0 |
| digital phantom | Run length matrix (3D, averaged) | Run entropy | 2,43 | 2,43207 | 0 | 0 |
| digital phantom | Run length matrix (3D, merged) | Short runs emphasis | 0,729 | 0,729127 | 0 | 0 |
| digital phantom | Run length matrix (3D, merged) | Long runs emphasis | 2,76 | 2,76147 | 0 | 0 |
| digital phantom | Run length matrix (3D, merged) | Low grey level run emphasis | 0,607 | 0,606651 | 0 | 0 |
| digital phantom | Run length matrix (3D, merged) | High grey level run emphasis | 9,64 | 9,63761 | 0 | 0 |
| digital phantom | Run length matrix (3D, merged) | Short run low grey level emphasis | 0,372 | 0,371603 | 0 | 0 |
| digital phantom | Run length matrix (3D, merged) | Short run high grey level emphasis | 8,67 | 8,67235 | 0 | 0 |
| digital phantom | Run length matrix (3D, merged) | Long run low grey level emphasis | 2,16 | 2,16287 | 0 | 0 |
| digital phantom | Run length matrix (3D, merged) | Long run high grey level emphasis | 15,6 | 15,6346 | 0 | 0 |
| digital phantom | Run length matrix (3D, merged) | Grey level non-uniformity | 281 | 281,281 | 0 | 0 |
| digital phantom | Run length matrix (3D, merged) | Grey level non-uniformity normalised | 0,43 | 0,430094 | 0 | 0 |
| digital phantom | Run length matrix (3D, merged) | Run length non-uniformity | 328 | 327,719 | 0 | 0 |
| digital phantom | Run length matrix (3D, merged) | Run length non-uniformity normalised | 0,501 | 0,501099 | 0 | 0 |
| digital phantom | Run length matrix (3D, merged) | Run percentage | 0,68 | 0,679834 | 0 | 0 |
| digital phantom | Run length matrix (3D, merged) | Grey level variance | 3,48 | 3,47902 | 0 | 0 |
| digital phantom | Run length matrix (3D, merged) | Run length variance | 0,598 | 0,59778 | 0 | 0 |
| digital phantom | Run length matrix (3D, merged) | Run entropy | 2,62 | 2,62443 | 0 | 0 |
| digital phantom | Size zone matrix (2D) | Small zone emphasis | 0,363 | 0,363308 | 0 | 0 |
| digital phantom | Size zone matrix (2D) | Large zone emphasis | 43,9 | 43,8667 | 0 | 0 |
| digital phantom | Size zone matrix (2D) | Low grey level emphasis | 0,371 | 0,371197 | 0 | 0 |
| digital phantom | Size zone matrix (2D) | High grey level emphasis | 16,4 | 16,4405 | 0 | 0 |
| digital phantom | Size zone matrix (2D) | Small zone low grey level emphasis | 0,0259 | 0,025855 | 0 | 0 |
| digital phantom | Size zone matrix (2D) | Small zone high grey level emphasis | 10,3 | 10,278 | 0 | 0 |
| digital phantom | Size zone matrix (2D) | Large zone low grey level emphasis | 40,4 | 40,3981 | 0 | 0 |
| digital phantom | Size zone matrix (2D) | Large zone high grey level emphasis | 113 | 112,521 | 0 | 0 |
| digital phantom | Size zone matrix (2D) | Grey level non-uniformity | 1,41 | 1,41429 | 0 | 0 |
| digital phantom | Size zone matrix (2D) | Grey level non uniformity normalised | 0,323 | 0,322993 | 0 | 0 |
| digital phantom | Size zone matrix (2D) | Zone size non-uniformity | 1,49 | 1,48571 | 0 | 0 |
| digital phantom | Size zone matrix (2D) | Zone size non-uniformity normalised | 0,333 | 0,333197 | 0 | 0 |
| digital phantom | Size zone matrix (2D) | Zone percentage | 0,24 | 0,24039 | 0 | 0 |
| digital phantom | Size zone matrix (2D) | Grey level variance | 3,97 | 3,96948 | 0 | 0 |
| digital phantom | Size zone matrix (2D) | Zone size variance | 21 | 20,9971 | 0 | 0 |
| digital phantom | Size zone matrix (2D) | Zone size entropy | 1,93 | 1,93194 | 0 | 0 |
| digital phantom | Size zone matrix (3D) | Small zone emphasis | 0,255 | 0,255182 | 0 | 0 |
| digital phantom | Size zone matrix (3D) | Large zone emphasis | 550 | 550 | 0 | 0 |
| digital phantom | Size zone matrix (3D) | Low grey level emphasis | 0,253 | 0,252778 | 0 | 0 |
| digital phantom | Size zone matrix (3D) | High grey level emphasis | 15,6 | 15,6 | 0 | 0 |
| digital phantom | Size zone matrix (3D) | Small zone low grey level emphasis | 0,0256 | 0,025604 | 0 | 0 |
| digital phantom | Size zone matrix (3D) | Small zone high grey level emphasis | 2,76 | 2,76335 | 0 | 0 |
| digital phantom | Size zone matrix (3D) | Large zone low grey level emphasis | 503 | 502,794 | 0 | 0 |
| digital phantom | Size zone matrix (3D) | Large zone high grey level emphasis | 1490 | 1494,6 | 0 | 0 |
| digital phantom | Size zone matrix (3D) | Grey level non-uniformity | 1,4 | 1,4 | 0 | 0 |
| digital phantom | Size zone matrix (3D) | Grey level non uniformity normalised | 0,28 | 0,28 | 0 | 0 |
| digital phantom | Size zone matrix (3D) | Zone size non-uniformity | 1 | 1 | 0 | 0 |
| digital phantom | Size zone matrix (3D) | Zone size non-uniformity normalised | 0,2 | 0,2 | 0 | 0 |
| digital phantom | Size zone matrix (3D) | Zone percentage | 0,0676 | 0,067568 | 0 | 0 |
| digital phantom | Size zone matrix (3D) | Grey level variance | 2,64 | 2,64 | 0 | 0 |
| digital phantom | Size zone matrix (3D) | Zone size variance | 331 | 330,96 | 0 | 0 |
| digital phantom | Size zone matrix (3D) | Zone size entropy | 2,32 | 2,32193 | 0 | 0 |
| digital phantom | Distance zone matrix (2D) | Small distance emphasis | 0,946 | 0,946429 | 0 | 0 |
| digital phantom | Distance zone matrix (2D) | Large distance emphasis | 1,21 | 1,21429 | 0 | 0 |
| digital phantom | Distance zone matrix (2D) | Low grey level emphasis | 0,371 | 0,371197 | 0 | 0 |
| digital phantom | Distance zone matrix (2D) | High grey level emphasis | 16,4 | 16,4405 | 0 | 0 |
| digital phantom | Distance zone matrix (2D) | Small distance low grey level emphasis | 0,367 | 0,367477 | 0 | 0 |
| digital phantom | Distance zone matrix (2D) | Small distance high grey level emphasis | 15,2 | 15,2351 | 0 | 0 |
| digital phantom | Distance zone matrix (2D) | Large distance low grey level emphasis | 0,386 | 0,386078 | 0 | 0 |
| digital phantom | Distance zone matrix (2D) | Large distance high grey level emphasis | 21,3 | 21,2619 | 0 | 0 |
| digital phantom | Distance zone matrix (2D) | Grey level non-uniformity | 1,41 | 1,41429 | 0 | 0 |
| digital phantom | Distance zone matrix (2D) | Grey level non-uniformity normalised | 0,323 | 0,322993 | 0 | 0 |
| digital phantom | Distance zone matrix (2D) | Zone distance non-uniformity | 3,79 | 3,78571 | 0 | 0 |
| digital phantom | Distance zone matrix (2D) | Zone distance non-uniformity normalised | 0,898 | 0,897959 | 0 | 0 |
| digital phantom | Distance zone matrix (2D) | Zone percentage | 0,24 | 0,24039 | 0 | 0 |
| digital phantom | Distance zone matrix (2D) | Grey level variance | 3,97 | 4,22222 | 0,25 | 6,297229 |
| digital phantom | Distance zone matrix (2D) | Zone distance variance | 0,051 | 0,05102 | 0 | 0 |
| digital phantom | Distance zone matrix (2D) | Zone distance entropy | 1,73 | 1,73194 | 0 | 0 |
| digital phantom | Distance zone matrix (3D) | Small distance emphasis | 1 | 1 | 0 | 0 |
| digital phantom | Distance zone matrix (3D) | Large distance emphasis | 1 | 1 | 0 | 0 |
| digital phantom | Distance zone matrix (3D) | Low grey level emphasis | 0,253 | 0,252778 | 0 | 0 |
| digital phantom | Distance zone matrix (3D) | High grey level emphasis | 15,6 | 15,6 | 0 | 0 |
| digital phantom | Distance zone matrix (3D) | Small distance low grey level emphasis | 0,253 | 0,252778 | 0 | 0 |
| digital phantom | Distance zone matrix (3D) | Small distance high grey level emphasis | 15,6 | 15,6 | 0 | 0 |
| digital phantom | Distance zone matrix (3D) | Large distance low grey level emphasis | 0,253 | 0,252778 | 0 | 0 |
| digital phantom | Distance zone matrix (3D) | Large distance high grey level emphasis | 15,6 | 15,6 | 0 | 0 |
| digital phantom | Distance zone matrix (3D) | Grey level non-uniformity | 1,4 | 1,4 | 0 | 0 |
| digital phantom | Distance zone matrix (3D) | Grey level non-uniformity normalised | 0,28 | 0,28 | 0 | 0 |
| digital phantom | Distance zone matrix (3D) | Zone distance non-uniformity | 5 | 5 | 0 | 0 |
| digital phantom | Distance zone matrix (3D) | Zone distance non-uniformity normalised | 1 | 1 | 0 | 0 |
| digital phantom | Distance zone matrix (3D) | Zone percentage | 0,0676 | 0,067568 | 0 | 0 |
| digital phantom | Distance zone matrix (3D) | Grey level variance | 2,64 | 2,64 | 0 | 0 |
| digital phantom | Distance zone matrix (3D) | Zone distance variance | 0 | 0 | 0 | 0 |
| digital phantom | Distance zone matrix (3D) | Zone distance entropy | 1,92 | 1,92193 | 0 | 0 |
| digital phantom | Neighbourhood grey tone difference matrix (2D) | Coarseness | 0,121 | 0,120511 | 0 | 0 |
| digital phantom | Neighbourhood grey tone difference matrix (2D) | Contrast | 0,925 | 0,925263 | 0 | 0 |
| digital phantom | Neighbourhood grey tone difference matrix (2D) | Busyness | 2,99 | 2,98879 | 0 | 0 |
| digital phantom | Neighbourhood grey tone difference matrix (2D) | Complexity | 10,4 | 10,4001 | 0 | 0 |
| digital phantom | Neighbourhood grey tone difference matrix (2D) | Strength | 2,88 | 2,87637 | 0 | 0 |
| digital phantom | Neighbourhood grey tone difference matrix (3D) | Coarseness | 0,0296 | 0,029604 | 0 | 0 |
| digital phantom | Neighbourhood grey tone difference matrix (3D) | Contrast | 0,584 | 0,583711 | 0 | 0 |
| digital phantom | Neighbourhood grey tone difference matrix (3D) | Busyness | 6,54 | 6,54357 | 0 | 0 |
| digital phantom | Neighbourhood grey tone difference matrix (3D) | Complexity | 13,5 | 13,5398 | 0 | 0 |
| digital phantom | Neighbourhood grey tone difference matrix (3D) | Strength | 0,763 | 0,763495 | 0 | 0 |
| digital phantom | Neighbouring grey level dependence matrix (2D) | Low dependence emphasis | 0,158 | 0,15807 | 0 | 0 |
| digital phantom | Neighbouring grey level dependence matrix (2D) | High dependence emphasis | 19,2 | 19,1738 | 0 | 0 |
| digital phantom | Neighbouring grey level dependence matrix (2D) | Low grey level count emphasis | 0,702 | 0,701753 | 0 | 0 |
| digital phantom | Neighbouring grey level dependence matrix (2D) | High grey level count emphasis | 7,49 | 7,48695 | 0 | 0 |
| digital phantom | Neighbouring grey level dependence matrix (2D) | Low dependence low grey level emphasis | 0,0473 | 0,047291 | 0 | 0 |
| digital phantom | Neighbouring grey level dependence matrix (2D) | Low dependence high grey level emphasis | 3,06 | 3,06491 | 0 | 0 |
| digital phantom | Neighbouring grey level dependence matrix (2D) | High dependence low grey level emphasis | 17,6 | 17,5997 | 0 | 0 |
| digital phantom | Neighbouring grey level dependence matrix (2D) | High dependence high grey level emphasis | 49,5 | 49,4777 | 0 | 0 |
| digital phantom | Neighbouring grey level dependence matrix (2D) | Grey level non-uniformity | 10,2 | 10,2464 | 0 | 0 |
| digital phantom | Neighbouring grey level dependence matrix (2D) | Grey level non-uniformity normalised | 0,562 | 0,561861 | 0 | 0 |
| digital phantom | Neighbouring grey level dependence matrix (2D) | Dependence count non-uniformity | 3,96 | 3,96465 | 0 | 0 |
| digital phantom | Neighbouring grey level dependence matrix (2D) | Dependence count non-uniformity normalised | 0,212 | 0,211772 | 0 | 0 |
| digital phantom | Neighbouring grey level dependence matrix (2D) | Dependence count percentage | 1 | 1 | 0 | 0 |
| digital phantom | Neighbouring grey level dependence matrix (2D) | Grey level variance | 2,7 | 2,70373 | 0 | 0 |
| digital phantom | Neighbouring grey level dependence matrix (2D) | Dependence count variance | 2,73 | 2,7295 | 0 | 0 |
| digital phantom | Neighbouring grey level dependence matrix (2D) | Dependence count entropy | 2,71 | 2,71429 | 0 | 0 |
| digital phantom | Neighbouring grey level dependence matrix (2D) | Dependence count energy | 0,17 | 0,170252 | 0 | 0 |
| digital phantom | Neighbouring grey level dependence matrix (3D) | Low dependence emphasis | 0,045 | 0,044996 | 0 | 0 |
| digital phantom | Neighbouring grey level dependence matrix (3D) | High dependence emphasis | 109 | 109 | 0 | 0 |
| digital phantom | Neighbouring grey level dependence matrix (3D) | Low grey level count emphasis | 0,693 | 0,693318 | 0 | 0 |
| digital phantom | Neighbouring grey level dependence matrix (3D) | High grey level count emphasis | 7,66 | 7,66216 | 0 | 0 |
| digital phantom | Neighbouring grey level dependence matrix (3D) | Low dependence low grey level emphasis | 0,00963 | 0,009631 | 0 | 0 |
| digital phantom | Neighbouring grey level dependence matrix (3D) | Low dependence high grey level emphasis | 0,736 | 0,736172 | 0 | 0 |
| digital phantom | Neighbouring grey level dependence matrix (3D) | High dependence low grey level emphasis | 102 | 102,451 | 0 | 0 |
| digital phantom | Neighbouring grey level dependence matrix (3D) | High dependence high grey level emphasis | 235 | 234,986 | 0 | 0 |
| digital phantom | Neighbouring grey level dependence matrix (3D) | Grey level non-uniformity | 37,9 | 37,9189 | 0 | 0 |
| digital phantom | Neighbouring grey level dependence matrix (3D) | Grey level non-uniformity normalised | 0,512 | 0,512418 | 0 | 0 |
| digital phantom | Neighbouring grey level dependence matrix (3D) | Dependence count non-uniformity | 4,86 | 4,86486 | 0 | 0 |
| digital phantom | Neighbouring grey level dependence matrix (3D) | Dependence count non-uniformity normalised | 0,0657 | 0,065741 | 0 | 0 |
| digital phantom | Neighbouring grey level dependence matrix (3D) | Dependence count percentage | 1 | 1 | 0 | 0 |
| digital phantom | Neighbouring grey level dependence matrix (3D) | Grey level variance | 3,05 | 3,04547 | 0 | 0 |
| digital phantom | Neighbouring grey level dependence matrix (3D) | Dependence count variance | 22,1 | 22,057 | 0 | 0 |
| digital phantom | Neighbouring grey level dependence matrix (3D) | Dependence count entropy | 4,4 | 4,40374 | 0 | 0 |
| digital phantom | Neighbouring grey level dependence matrix (3D) | Dependence count energy | 0,0533 | 0,053324 | 0 | 0 |
