# Supplementary material for: RaCaT: An open source and easy to use radiomics calculator tool
Source: PLoS One. 2019 Feb 20;14(2):e0212223. doi: 10.1371/journal.pone.0212223 (PMC6382170; doi:10.1371/journal.pone.0212223)
Supplement: S2 Table — Benchmark feature values and values calculated by RaCaT as well as their differences and percentage differences for the realistic phantom, config A provided by IBSI. (DOCX) [file pone.0212223.s003.docx]

S Table 2: Benchmark feature values and values calculated by RaCaT as well as their differences and percentage differences for the realistic phantom. config A provided by IBSI

| **data_set** | **family** | **image_biomarker** | **benchmark_value** | **RaCaT** | **difference** | **%difference** |
| --- | --- | --- | --- | --- | --- | --- |
| configuration A | Morphology | Volume (mesh-based) | 358000 | 358681 | 0 | 0 |
| configuration A | Morphology | Volume (counting) | 359000 | 358681 | 0 | 0 |
| configuration A | Morphology | Surface area | 35700 | 33206,9 | 2400 | 6,722689 |
| configuration A | Morphology | Surface to volume ratio | 0,0996 | 0,09258 | 0,007 | 7,028112 |
| configuration A | Morphology | Compactness 1 | 0,03 | 0,033442 | 0,0034 | 11,33333 |
| configuration A | Morphology | Compactness 2 | 0,319 | 0,397362 | 0,078 | 24,45141 |
| configuration A | Morphology | Spherical disproportion | 1,46 | 1,3602 | 0,09 | 6,164384 |
| configuration A | Morphology | Sphericity | 0,683 | 0,735183 | 0,052 | 7,61347 |
| configuration A | Morphology | Asphericity | 0,463 | 0,360201 | 0,102 | 22,03024 |
| configuration A | Morphology | Centre of mass shift | 52,9 | 53,7972 | 0,8 | 1,512287 |
| configuration A | Morphology | Maximum 3D diameter | 125 | 123,354 | 1 | 0,8 |
| configuration A | Morphology | Major axis length | 92,7 | 92,7206 | 0 | 0 |
| configuration A | Morphology | Minor axis length | 81,5 | 81,5229 | 0 | 0 |
| configuration A | Morphology | Least axis length | 70,1 | 70,0632 | 0 | 0 |
| configuration A | Morphology | Elongation | 0,879 | 0,937674 | 0,058 | 6,598407 |
| configuration A | Morphology | Flatness | 0,756 | 0,869274 | 0,113 | 14,94709 |
| configuration A | Morphology | Volume density (AEE) | 1,29 | 1,29 | 0 | 0 |
| configuration A | Local intensity | Local intensity peak | -277 | -270,434 | 6 | -2,16606 |
| configuration A | Local intensity | Global intensity peak | 189 | 195,935 | 6 | 3,174603 |
| configuration A | Statistics | Mean | 13,4 | 13,4084 | 0 | 0 |
| configuration A | Statistics | Variance | 14200 | 14233,2 | 0 | 0 |
| configuration A | Statistics | Skewness | -2,47 | -2,47363 | 0 | 0 |
| configuration A | Statistics | (Excess) kurtosis | 5,96 | 5,95599 | 0 | 0 |
| configuration A | Statistics | Median | 46 | 46 | 0 | 0 |
| configuration A | Statistics | Minimum | -500 | -500 | 0 | 0 |
| configuration A | Statistics | 10th percentile | -129 | -129 | 0 | 0 |
| configuration A | Statistics | 90th percentile | 95 | 93 | 2 | 2,105263 |
| configuration A | Statistics | Maximum | 377 | 377 | 0 | 0 |
| configuration A | Statistics | Interquartile range | 56 | 56 | 0 | 0 |
| configuration A | Statistics | Range | 877 | 877 | 0 | 0 |
| configuration A | Statistics | Mean absolute deviation | 73,6 | 73,5607 | 0 | 0 |
| configuration A | Statistics | Robust mean absolute deviation | 27,7 | 27,577 | 0,1 | 0,361011 |
| configuration A | Statistics | Median absolute deviation | 64,3 | 64,2906 | 0 | 0 |
| configuration A | Statistics | Coefficient of variation | 8,9 | 8,89761 | 0 | 0 |
| configuration A | Statistics | Quartile coefficient of dispersion | 0,636 | 0,636364 | 0 | 0 |
| configuration A | Statistics | Energy | 1,65E+09 | 1,65E+09 | 0 | 0 |
| configuration A | Statistics | Root mean square | 120 | 120,06 | 0 | 0 |
| configuration A | Intensity histogram | Mean | 21 | 21,0571 | 0 | 0 |
| configuration A | Intensity histogram | Variance | 22,8 | 22,814 | 0 | 0 |
| configuration A | Intensity histogram | Skewness | -2,46 | -2,33878 | 0,12 | -4,87805 |
| configuration A | Intensity histogram | Kurtosis | 5,9 | 4,89643 | 1 | 16,94915 |
| configuration A | Intensity histogram | Median | 22 | 22 | 0 | 0 |
| configuration A | Intensity histogram | Minimum | 1 | 1 | 0 | 0 |
| configuration A | Intensity histogram | 10th percentile | 15 | 15 | 0 | 0 |
| configuration A | Intensity histogram | 90th percentile | 24 | 24 | 0 | 0 |
| configuration A | Intensity histogram | Maximum | 36 | 36 | 0 | 0 |
| configuration A | Intensity histogram | Mode | 23 | 21 | 2 | 8,695652 |
| configuration A | Intensity histogram | Interquartile range | 2 | 2 | 0 | 0 |
| configuration A | Intensity histogram | Range | 35 | 35 | 0 | 0 |
| configuration A | Intensity histogram | Mean absolute deviation | 2,93 | 2,93942 | 0 | 0 |
| configuration A | Intensity histogram | Robust mean absolute deviation | 1,18 | 1,2359 | 0,05 | 4,237288 |
| configuration A | Intensity histogram | Median absolute deviation | 2,57 | 2,58026 | 0,01 | 0,389105 |
| configuration A | Intensity histogram | Coefficient of variation | 0,227 | 0,226831 | 0 | 0 |
| configuration A | Intensity histogram | Quartile coefficient of dispersion | 0,0455 | 0,045455 | 0 | 0 |
| configuration A | Intensity histogram | Entropy | 3,36 | 3,35596 | 0 | 0 |
| configuration A | Intensity histogram | Uniformity | 0,15 | 0,150375 | 0 | 0 |
| configuration A | Intensity histogram | Maximum histogram gradient | 11000 | 11039,5 | 0 | 0 |
| configuration A | Intensity histogram | Maximum gradient grey level | 21 | 21 | 0 | 0 |
| configuration A | Intensity histogram | Minimum histogram gradient | -10100 | -10101,5 | 0 | 0 |
| configuration A | Intensity histogram | Minimum gradient grey level | 24 | 24 | 0 | 0 |
| configuration A | Intensity volume histogram | Volume fraction at 10% intensity | 0,978 | 0,978193 |  | 0 |
| configuration A | Intensity volume histogram | Volume fraction at 90% intensity | 6,98E-05 | 6,98E-05 | 0 | 0 |
| configuration A | Intensity volume histogram | Intensity at 10% volume | 96 | 96 | 0 | 0 |
| configuration A | Intensity volume histogram | Intensity at 90% volume | -128 | -128 | 0 | 0 |
| configuration A | Intensity volume histogram | Volume fraction difference between 10% and 90% intensity | 0,978 | 0,978123 | 0 | 0 |
| configuration A | Intensity volume histogram | Intensity difference between 10% and 90% volume | 224 | 224 | 0 | 0 |
| configuration A | Co-occurrence matrix (2D, averaged) | Joint maximum | 0,109 | 0,108895 | 0 | 0 |
| configuration A | Co-occurrence matrix (2D, averaged) | Joint average | 20,6 | 20,6485 | 0 | 0 |
| configuration A | Co-occurrence matrix (2D, averaged) | Joint variance | 27 | 27,0052 | 0 | 0 |
| configuration A | Co-occurrence matrix (2D, averaged) | Joint entropy | 5,82 | 5,81992 | 0 | 0 |
| configuration A | Co-occurrence matrix (2D, averaged) | Difference average | 1,58 | 1,57645 | 0 | 0 |
| configuration A | Co-occurrence matrix (2D, averaged) | Difference variance | 4,94 | 4,93623 | 0 | 0 |
| configuration A | Co-occurrence matrix (2D, averaged) | Difference entropy | 2,27 | 2,27041 | 0 | 0 |
| configuration A | Co-occurrence matrix (2D, averaged) | Sum average | 41,3 | 41,297 | 0 | 0 |
| configuration A | Co-occurrence matrix (2D, averaged) | Sum variance | 100 | 100,174 | 0 | 0 |
| configuration A | Co-occurrence matrix (2D, averaged) | Sum entropy | 4,19 | 4,19054 | 0 | 0 |
| configuration A | Co-occurrence matrix (2D, averaged) | Angular second moment | 0,045 | 0,044951 | 0 | 0 |
| configuration A | Co-occurrence matrix (2D, averaged) | Contrast | 7,85 | 7,84693 | 0 | 0 |
| configuration A | Co-occurrence matrix (2D, averaged) | Dissimilarity | 1,58 | 1,57645 | 0 | 0 |
| configuration A | Co-occurrence matrix (2D, averaged) | Inverse difference | 0,581 | 0,581088 | 0 | 0 |
| configuration A | Co-occurrence matrix (2D, averaged) | Inverse difference normalised | 0,961 | 0,961129 | 0 | 0 |
| configuration A | Co-occurrence matrix (2D, averaged) | Inverse difference moment | 0,544 | 0,543808 | 0 | 0 |
| configuration A | Co-occurrence matrix (2D, averaged) | Inverse difference moment normalised | 0,994 | 0,994371 | 0 | 0 |
| configuration A | Co-occurrence matrix (2D, averaged) | Inverse variance | 0,441 | 0,441078 | 0 | 0 |
| configuration A | Co-occurrence matrix (2D, averaged) | Correlation | 0,778 | 0,77798 | 0 | 0 |
| configuration A | Co-occurrence matrix (2D, averaged) | Autocorrelation | 455 | 455,372 | 0 | 0 |
| configuration A | Co-occurrence matrix (2D, averaged) | Cluster tendency | 100 | 100,174 | 0 | 0 |
| configuration A | Co-occurrence matrix (2D, averaged) | Cluster shade | -1040 | -1042,14 | 0 | 0 |
| configuration A | Co-occurrence matrix (2D, averaged) | Cluster prominence | 52700 | 52624,2 | 0 | 0 |
| configuration A | Co-occurrence matrix (2D, averaged) | Information correlation 1 | -0,236 | -0,2361 | 0 | 0 |
| configuration A | Co-occurrence matrix (2D, averaged) | Information correlation 2 | 0,863 | 0,863442 | 0 | 0 |
| configuration A | Co-occurrence matrix (2D, slice-merged) | Joint maximum | 0,109 | 0,108756 | 0 | 0 |
| configuration A | Co-occurrence matrix (2D, slice-merged) | Joint average | 20,6 | 20,6468 | 0 | 0 |
| configuration A | Co-occurrence matrix (2D, slice-merged) | Joint variance | 27 | 27,0283 | 0 | 0 |
| configuration A | Co-occurrence matrix (2D, slice-merged) | Joint entropy | 5,9 | 5,90265 | 0 | 0 |
| configuration A | Co-occurrence matrix (2D, slice-merged) | Difference average | 1,57 | 1,57411 | 0 | 0 |
| configuration A | Co-occurrence matrix (2D, slice-merged) | Difference variance | 4,96 | 4,95964 | 0 | 0 |
| configuration A | Co-occurrence matrix (2D, slice-merged) | Difference entropy | 2,28 | 2,2833 | 0 | 0 |
| configuration A | Co-occurrence matrix (2D, slice-merged) | Sum average | 41,3 | 41,2936 | 0 | 0 |
| configuration A | Co-occurrence matrix (2D, slice-merged) | Sum variance | 100 | 100,295 | 0 | 0 |
| configuration A | Co-occurrence matrix (2D, slice-merged) | Sum entropy | 4,21 | 4,20658 | 0 | 0 |
| configuration A | Co-occurrence matrix (2D, slice-merged) | Angular second moment | 0,0446 | 0,044645 | 0 | 0 |
| configuration A | Co-occurrence matrix (2D, slice-merged) | Contrast | 7,82 | 7,81817 | 0 | 0 |
| configuration A | Co-occurrence matrix (2D, slice-merged) | Dissimilarity | 1,57 | 1,57411 | 0 | 0 |
| configuration A | Co-occurrence matrix (2D, slice-merged) | Inverse difference | 0,581 | 0,581258 | 0 | 0 |
| configuration A | Co-occurrence matrix (2D, slice-merged) | Inverse difference normalised | 0,961 | 0,961178 | 0 | 0 |
| configuration A | Co-occurrence matrix (2D, slice-merged) | Inverse difference moment | 0,544 | 0,544006 | 0 | 0 |
| configuration A | Co-occurrence matrix (2D, slice-merged) | Inverse difference moment normalised | 0,994 | 0,99439 | 0 | 0 |
| configuration A | Co-occurrence matrix (2D, slice-merged) | Inverse variance | 0,441 | 0,441227 | 0 | 0 |
| configuration A | Co-occurrence matrix (2D, slice-merged) | Correlation | 0,78 | 0,780247 | 0 | 0 |
| configuration A | Co-occurrence matrix (2D, slice-merged) | Autocorrelation | 455 | 455,334 | 0 | 0 |
| configuration A | Co-occurrence matrix (2D, slice-merged) | Cluster tendency | 100 | 100,295 | 0 | 0 |
| configuration A | Co-occurrence matrix (2D, slice-merged) | Cluster shade | -1050 | -1044,86 | 0 | 0 |
| configuration A | Co-occurrence matrix (2D, slice-merged) | Cluster prominence | 52800 | 52723,5 | 0 | 0 |
| configuration A | Co-occurrence matrix (2D, slice-merged) | Information correlation 1 | -0,214 | -0,21357 | 0 | 0 |
| configuration A | Co-occurrence matrix (2D, slice-merged) | Information correlation 2 | 0,851 | 0,851016 | 0 | 0 |
| configuration A | Run length matrix (2D, averaged) | Short runs emphasis | 0,785 | 0,785002 | 0 | 0 |
| configuration A | Run length matrix (2D, averaged) | Long runs emphasis | 2,91 | 2,90545 | 0 | 0 |
| configuration A | Run length matrix (2D, averaged) | Low grey level run emphasis | 0,0264 | 0,026416 | 0 | 0 |
| configuration A | Run length matrix (2D, averaged) | High grey level run emphasis | 428 | 428,196 | 0 | 0 |
| configuration A | Run length matrix (2D, averaged) | Short run low grey level emphasis | 0,0243 | 0,024332 | 0 | 0 |
| configuration A | Run length matrix (2D, averaged) | Short run high grey level emphasis | 320 | 320,049 | 0 | 0 |
| configuration A | Run length matrix (2D, averaged) | Long run low grey level emphasis | 0,0386 | 0,038556 | 0 | 0 |
| configuration A | Run length matrix (2D, averaged) | Long run high grey level emphasis | 1400 | 1405,94 | 0 | 0 |
| configuration A | Run length matrix (2D, averaged) | Grey level non-uniformity | 432 | 432,121 | 0 | 0 |
| configuration A | Run length matrix (2D, averaged) | Grey level non-uniformity normalised | 0,128 | 0,12842 | 0 | 0 |
| configuration A | Run length matrix (2D, averaged) | Run length non-uniformity | 1650 | 1653,93 | 0 | 0 |
| configuration A | Run length matrix (2D, averaged) | Run length non-uniformity normalised | 0,579 | 0,578645 | 0 | 0 |
| configuration A | Run length matrix (2D, averaged) | Run percentage | 0,704 | 0,704147 | 0 | 0 |
| configuration A | Run length matrix (2D, averaged) | Grey level variance | 33,6 | 33,6905 | 0 | 0 |
| configuration A | Run length matrix (2D, averaged) | Run length variance | 0,828 | 0,828163 | 0 | 0 |
| configuration A | Run length matrix (2D, averaged) | Run entropy | 4,73 | 4,73472 | 0 | 0 |
| configuration A | Run length matrix (2D, slice-merged) | Short runs emphasis | 0,786 | 0,785819 | 0 | 0 |
| configuration A | Run length matrix (2D, slice-merged) | Long runs emphasis | 2,89 | 2,89337 | 0 | 0 |
| configuration A | Run length matrix (2D, slice-merged) | Low grey level run emphasis | 0,0264 | 0,026406 | 0 | 0 |
| configuration A | Run length matrix (2D, slice-merged) | High grey level run emphasis | 428 | 428,251 | 0 | 0 |
| configuration A | Run length matrix (2D, slice-merged) | Short run low grey level emphasis | 0,0243 | 0,02433 | 0 | 0 |
| configuration A | Run length matrix (2D, slice-merged) | Short run high grey level emphasis | 320 | 320,496 | 0 | 0 |
| configuration A | Run length matrix (2D, slice-merged) | Long run low grey level emphasis | 0,0385 | 0,038469 | 0 | 0 |
| configuration A | Run length matrix (2D, slice-merged) | Long run high grey level emphasis | 1400 | 1399,97 | 0 | 0 |
| configuration A | Run length matrix (2D, slice-merged) | Grey level non-uniformity | 1730 | 1728,02 | 0 | 0 |
| configuration A | Run length matrix (2D, slice-merged) | Grey level non-uniformity normalised | 0,128 | 0,128406 | 0 | 0 |
| configuration A | Run length matrix (2D, slice-merged) | Run length non-uniformity | 6600 | 6603,71 | 0 | 0 |
| configuration A | Run length matrix (2D, slice-merged) | Run length non-uniformity normalised | 0,579 | 0,578887 | 0 | 0 |
| configuration A | Run length matrix (2D, slice-merged) | Run percentage | 0,704 | 0,704147 | 0 | 0 |
| configuration A | Run length matrix (2D, slice-merged) | Grey level variance | 33,7 | 33,6851 | 0 | 0 |
| configuration A | Run length matrix (2D, slice-merged) | Run length variance | 0,826 | 0,825965 | 0 | 0 |
| configuration A | Run length matrix (2D, slice-merged) | Run entropy | 4,76 | 4,75675 | 0 | 0 |
| configuration A | Size zone matrix (2D) | Small zone emphasis | 0,688 | 0,687965 | 0 | 0 |
| configuration A | Size zone matrix (2D) | Large zone emphasis | 625 | 625,368 | 0 | 0 |
| configuration A | Size zone matrix (2D) | Low grey level emphasis | 0,0368 | 0,036582 | 0,0002 | 0,543478 |
| configuration A | Size zone matrix (2D) | High grey level emphasis | 363 | 363,244 | 0 | 0 |
| configuration A | Size zone matrix (2D) | Small zone low grey level emphasis | 0,0298 | 0,029785 | 0 | 0 |
| configuration A | Size zone matrix (2D) | Small zone high grey level emphasis | 226 | 226,138 | 0 | 0 |
| configuration A | Size zone matrix (2D) | Large zone low grey level emphasis | 1,35 | 1,34759 | 0 | 0 |
| configuration A | Size zone matrix (2D) | Large zone high grey level emphasis | 316000 | 315492 | 0 | 0 |
| configuration A | Size zone matrix (2D) | Grey level non-uniformity | 82,2 | 82,2577 | 0 | 0 |
| configuration A | Size zone matrix (2D) | Grey level non uniformity normalised | 0,0728 | 0,072849 | 0 | 0 |
| configuration A | Size zone matrix (2D) | Zone size non-uniformity | 479 | 478,991 | 0 | 0 |
| configuration A | Size zone matrix (2D) | Zone size non-uniformity normalised | 0,44 | 0,440731 | 0 | 0 |
| configuration A | Size zone matrix (2D) | Zone percentage | 0,3 | 0,300308 | 0 | 0 |
| configuration A | Size zone matrix (2D) | Grey level variance | 42,7 | 42,7224 | 0 | 0 |
| configuration A | Size zone matrix (2D) | Zone size variance | 609 | 609,38 | 0 | 0 |
| configuration A | Size zone matrix (2D) | Zone size entropy | 5,92 | 5,92079 | 0 | 0 |
| configuration A | Distance zone matrix (2D) | Small distance emphasis | 0,191 | 0,191849 | 0 | 0 |
| configuration A | Distance zone matrix (2D) | Large distance emphasis | 162 | 164,228 | 2 | 1,234568 |
| configuration A | Distance zone matrix (2D) | Low grey level emphasis | 0,0376 | 0,036792 | 0,0008 | 2,12766 |
| configuration A | Distance zone matrix (2D) | High grey level emphasis | 362 | 363,337 | 1 | 0,276243 |
| configuration A | Distance zone matrix (2D) | Small distance low grey level emphasis | 0,00942 | 0,009116 | 0,0003 | 3,184713 |
| configuration A | Distance zone matrix (2D) | Small distance high grey level emphasis | 60,1 | 59,9637 | 0,1 | 0,166389 |
| configuration A | Distance zone matrix (2D) | Large distance low grey level emphasis | 2,97 | 3,08208 | 0,11 | 3,703704 |
| configuration A | Distance zone matrix (2D) | Large distance high grey level emphasis | 70500 | 71429,7 | 900 | 1,276596 |
| configuration A | Distance zone matrix (2D) | Grey level non-uniformity | 82,6 | 82,2339 | 0,3 | 0,363196 |
| configuration A | Distance zone matrix (2D) | Grey level non-uniformity normalised | 0,0728 | 0,072835 | 0 | 0 |
| configuration A | Distance zone matrix (2D) | Zone distance non-uniformity | 63,5 | 63,2503 | 0,2 | 0,314961 |
| configuration A | Distance zone matrix (2D) | Zone distance non-uniformity normalised | 0,0716 | 0,07089 | 0,0007 | 0,977654 |
| configuration A | Distance zone matrix (2D) | Zone percentage | 0,299 | 0,300075 | 0,001 | 0,334448 |
| configuration A | Distance zone matrix (2D) | Grey level variance | 42,6 | 30,4363 | 12,1 | 28,40376 |
| configuration A | Distance zone matrix (2D) | Zone distance variance | 69,7 | 70,7701 | 1 | 1,43472 |
| configuration A | Distance zone matrix (2D) | Zone distance entropy | 8 | 8,01109 | 0,01 | 0,125 |
| configuration A | Neighbourhood grey tone difference matrix (2D) | Coarseness | 0,00629 | 0,006282 | 0 | 0 |
| configuration A | Neighbourhood grey tone difference matrix (2D) | Contrast | 0,107 | 0,107566 | 0 | 0 |
| configuration A | Neighbourhood grey tone difference matrix (2D) | Busyness | 0,489 | 0,489135 | 0 | 0 |
| configuration A | Neighbourhood grey tone difference matrix (2D) | Complexity | 438 | 438,228 | 0 | 0 |
| configuration A | Neighbourhood grey tone difference matrix (2D) | Strength | 3,33 | 3,32489 | 0 | 0 |
| configuration A | Neighbouring grey level dependence matrix (2D) | Low dependence emphasis | 0,281 | 0,281491 | 0 | 0 |
| configuration A | Neighbouring grey level dependence matrix (2D) | High dependence emphasis | 14,8 | 14,757 | 0 | 0 |
| configuration A | Neighbouring grey level dependence matrix (2D) | Low grey level count emphasis | 0,0233 | 0,023299 | 0 | 0 |
| configuration A | Neighbouring grey level dependence matrix (2D) | High grey level count emphasis | 446 | 446,193 | 0 | 0 |
| configuration A | Neighbouring grey level dependence matrix (2D) | Low dependence low grey level emphasis | 0,0137 | 0,013723 | 0 | 0 |
| configuration A | Neighbouring grey level dependence matrix (2D) | Low dependence high grey level emphasis | 94,2 | 94,2438 | 0 | 0 |
| configuration A | Neighbouring grey level dependence matrix (2D) | High dependence low grey level emphasis | 0,116 | 0,115847 | 0 | 0 |
| configuration A | Neighbouring grey level dependence matrix (2D) | High dependence high grey level emphasis | 7540 | 7539,79 | 0 | 0 |
| configuration A | Neighbouring grey level dependence matrix (2D) | Grey level non-uniformity | 757 | 757,261 | 0 | 0 |
| configuration A | Neighbouring grey level dependence matrix (2D) | Grey level non-uniformity normalised | 0,151 | 0,151417 | 0 | 0 |
| configuration A | Neighbouring grey level dependence matrix (2D) | Dependence count non-uniformity | 709 | 708,785 | 0 | 0 |
| configuration A | Neighbouring grey level dependence matrix (2D) | Dependence count non-uniformity normalised | 0,175 | 0,175293 | 0 | 0 |
| configuration A | Neighbouring grey level dependence matrix (2D) | Dependence count percentage | 1 | 1 | 0 | 0 |
| configuration A | Neighbouring grey level dependence matrix (2D) | Grey level variance | 31,1 | 31,1051 | 0 | 0 |
| configuration A | Neighbouring grey level dependence matrix (2D) | Dependence count variance | 3,12 | 3,12442 | 0 | 0 |
| configuration A | Neighbouring grey level dependence matrix (2D) | Dependence count entropy | 5,76 | 5,76073 | 0 | 0 |
| configuration A | Neighbouring grey level dependence matrix (2D) | Dependence count energy | 0,0268 | 0,026832 | 0 | 0 |
