# Supplementary material for: RaCaT: An open source and easy to use radiomics calculator tool
Source: PLoS One. 2019 Feb 20;14(2):e0212223. doi: 10.1371/journal.pone.0212223 (PMC6382170; doi:10.1371/journal.pone.0212223)
Supplement: S3 Table — Benchmark feature values and values calculated by RaCaT as well as their differences and percentage differences for the realistic phantom, config C provided by IBSI. (DOCX) [file pone.0212223.s004.docx]

S3 Table: Benchmark feature values and values calculated by RaCaT as well as their differences and percentage differences for the realistic phantom. config C provided by IBSI

| **data_set** | **family** | **image_biomarker** | **benchmark_value** | **your_result** | **difference** | **%difference** |
| --- | --- | --- | --- | --- | --- | --- |
| configuration C | Morphology | Volume (mesh-based) | 367000 | 367880 | 0 | 0 |
| configuration C | Morphology | Volume (counting) | 368000 | 367880 | 0 | 0 |
| configuration C | Morphology | Surface area | 34300 | 31556,9 | 2700 | 7,87172 |
| configuration C | Morphology | Surface to volume ratio | 0,0934 | 0,08578 | 0,0076 | 8,137045 |
| configuration C | Morphology | Compactness 1 | 0,0326 | 0,037025 | 0,0044 | 13,49693 |
| configuration C | Morphology | Compactness 2 | 0,378 | 0,487059 | 0,109 | 28,83598 |
| configuration C | Morphology | Spherical disproportion | 1,38 | 1,27098 | 0,1 | 7,246377 |
| configuration C | Morphology | Sphericity | 0,723 | 0,786793 | 0,063 | 8,713693 |
| configuration C | Morphology | Asphericity | 0,383 | 0,270979 | 0,112 | 29,24282 |
| configuration C | Morphology | Centre of mass shift | 45,6 | 45,5624 | 0 | 0 |
| configuration C | Morphology | Maximum 3D diameter | 125 | 123,726 | 1 | 0,8 |
| configuration C | Morphology | Major axis length | 93,3 | 93,2693 | 0 | 0 |
| configuration C | Morphology | Minor axis length | 82 | 82,0043 | 0 | 0 |
| configuration C | Morphology | Least axis length | 70,9 | 70,9008 | 0 | 0 |
| configuration C | Morphology | Elongation | 0,879 | 0,937667 | 0,058 | 6,598407 |
| configuration C | Morphology | Flatness | 0,76 | 0,871879 | 0,111 | 14,60526 |
| configuration C | Morphology | Volume density (AABB) | 0,478 | 0,478811 | 0 | 0 |
| configuration C | Morphology | Area density (AABB) | 0,678 | 0,624049 | 0,053 | 7,817109 |
| configuration C | Morphology | Volume density (AEE) | 1,29 | 1,29563 | 0 | 0 |
| configuration C | Morphology | Area density (AEE) | 1,62 | 1,49 | 0,13 | 8,024691 |
| configuration C | Morphology | Integrated intensity | -1,8E+07 | -1,80E+07 | 0 | 0 |
| configuration C | Morphology | Moran's I index | 0,0824 | 0,056298 | 0,0261 | 31,67476 |
| configuration C | Morphology | Geary's C measure | 0,846 | 0,862128 | 0,016 | 1,891253 |
| configuration C | Local intensity | Local intensity peak | 169 | 199 | 30 | 17,75148 |
| configuration C | Local intensity | Global intensity peak | 180 | 210 | 30 | 16,66667 |
| configuration C | Statistics | Mean | -49 | -48,9785 | 0 | 0 |
| configuration C | Statistics | Variance | 50600 | 50639,5 | 0 | 0 |
| configuration C | Statistics | Skewness | -2,14 | -2,14016 | 0 | 0 |
| configuration C | Statistics | (Excess) kurtosis | 3,53 | 3,52481 | 0 | 0 |
| configuration C | Statistics | Median | 40 | 40 | 0 | 0 |
| configuration C | Statistics | Minimum | -939 | -939 | 0 | 0 |
| configuration C | Statistics | 10th percentile | -424 | -424 | 0 | 0 |
| configuration C | Statistics | 90th percentile | 86 | 84 | 2 | 2,325581 |
| configuration C | Statistics | Maximum | 393 | 393 | 0 | 0 |
| configuration C | Statistics | Interquartile range | 67 | 67 | 0 | 0 |
| configuration C | Statistics | Range | 1330 | 1332 | 0 | 0 |
| configuration C | Statistics | Mean absolute deviation | 158 | 157,983 | 0 | 0 |
| configuration C | Statistics | Robust mean absolute deviation | 66,8 | 67,4041 | 0,6 | 0,898204 |
| configuration C | Statistics | Median absolute deviation | 119 | 119,127 | 0 | 0 |
| configuration C | Statistics | Coefficient of variation | -4,59 | -4,59451 | 0 | 0 |
| configuration C | Statistics | Quartile coefficient of dispersion | 1,03 | 1,03077 | 0 | 0 |
| configuration C | Statistics | Energy | 2,44E+09 | 2,44E+09 | 0 | 0 |
| configuration C | Statistics | Root mean square | 230 | 230,302 | 0 | 0 |
| configuration C | Intensity histogram | Mean | 38,6 | 38,5583 | 0 | 0 |
| configuration C | Intensity histogram | Variance | 81,1 | 81,1118 | 0 | 0 |
| configuration C | Intensity histogram | Skewness | -2,14 | -2,08771 | 0,05 | -2,33645 |
| configuration C | Intensity histogram | Kurtosis | 3,52 | 3,11737 | 0,4 | 11,36364 |
| configuration C | Intensity histogram | Median | 42 | 42 | 0 | 0 |
| configuration C | Intensity histogram | Minimum | 3 | 3 | 0 | 0 |
| configuration C | Intensity histogram | 10th percentile | 24 | 24 | 0 | 0 |
| configuration C | Intensity histogram | 90th percentile | 44 | 44 | 0 | 0 |
| configuration C | Intensity histogram | Maximum | 56 | 56 | 0 | 0 |
| configuration C | Intensity histogram | Mode | 43 | 43 | 0 | 0 |
| configuration C | Intensity histogram | Interquartile range | 3 | 3 | 0 | 0 |
| configuration C | Intensity histogram | Range | 53 | 53 | 0 | 0 |
| configuration C | Intensity histogram | Mean absolute deviation | 6,32 | 6,32164 | 0 | 0 |
| configuration C | Intensity histogram | Robust mean absolute deviation | 2,59 | 2,76045 | 0,17 | 6,563707 |
| configuration C | Intensity histogram | Median absolute deviation | 4,75 | 4,75038 | 0 | 0 |
| configuration C | Intensity histogram | Coefficient of variation | 0,234 | 0,233574 | 0 | 0 |
| configuration C | Intensity histogram | Quartile coefficient of dispersion | 0,0361 | 0,036145 | 0 | 0 |
| configuration C | Intensity histogram | Entropy | 3,73 | 3,73448 | 0 | 0 |
| configuration C | Intensity histogram | Uniformity | 0,14 | 0,139547 | 0 | 0 |
| configuration C | Intensity histogram | Maximum histogram gradient | 4750 | 4745,5 | 0 | 0 |
| configuration C | Intensity histogram | Maximum gradient grey level | 41 | 42 | 1 | 2,439024 |
| configuration C | Intensity histogram | Minimum histogram gradient | -4680 | -4677 | 0 | 0 |
| configuration C | Intensity histogram | Minimum gradient grey level | 44 | 45 | 1 | 2,272727 |
| configuration C | Intensity volume histogram | Volume fraction at 10% intensity | 0,998 | 0,987691 | 0,01 | 1,002004 |
| configuration C | Intensity volume histogram | Volume fraction at 90% intensity | 0,000152 | 0,000152 | 0 | 0 |
| configuration C | Intensity volume histogram | Intensity at 10% volume | 88,8 | 544 | 455,2 | 512,6126 |
| configuration C | Intensity volume histogram | Intensity at 90% volume | -421 | 289 | 710 | -168,646 |
| configuration C | Intensity volume histogram | Volume fraction difference between 10% and 90% intensity | 0,997 | 0,987408 | 0,009 | 0,902708 |
| configuration C | Intensity volume histogram | Intensity difference between 10% and 90% volume | 510 | 255 | 255 | 50 |
| configuration C | Co-occurrence matrix (3D, averaged) | Joint maximum | 0,111 | 0,110849 | 0 | 0 |
| configuration C | Co-occurrence matrix (3D, averaged) | Joint average | 39 | 38,9779 | 0 | 0 |
| configuration C | Co-occurrence matrix (3D, averaged) | Joint variance | 73,7 | 73,745 | 0 | 0 |
| configuration C | Co-occurrence matrix (3D, averaged) | Joint entropy | 6,39 | 6,38943 | 0 | 0 |
| configuration C | Co-occurrence matrix (3D, averaged) | Difference average | 2,17 | 2,16716 | 0 | 0 |
| configuration C | Co-occurrence matrix (3D, averaged) | Difference variance | 14,4 | 14,3781 | 0 | 0 |
| configuration C | Co-occurrence matrix (3D, averaged) | Difference entropy | 2,64 | 2,63542 | 0 | 0 |
| configuration C | Co-occurrence matrix (3D, averaged) | Sum average | 78 | 77,9559 | 0 | 0 |
| configuration C | Co-occurrence matrix (3D, averaged) | Sum variance | 276 | 275,803 | 0 | 0 |
| configuration C | Co-occurrence matrix (3D, averaged) | Sum entropy | 4,56 | 4,5556 | 0 | 0 |
| configuration C | Co-occurrence matrix (3D, averaged) | Angular second moment | 0,045 | 0,045002 | 0 | 0 |
| configuration C | Co-occurrence matrix (3D, averaged) | Contrast | 19,2 | 19,1775 | 0 | 0 |
| configuration C | Co-occurrence matrix (3D, averaged) | Dissimilarity | 2,17 | 2,16716 | 0 | 0 |
| configuration C | Co-occurrence matrix (3D, averaged) | Inverse difference | 0,582 | 0,582396 | 0 | 0 |
| configuration C | Co-occurrence matrix (3D, averaged) | Inverse difference normalised | 0,966 | 0,966193 | 0 | 0 |
| configuration C | Co-occurrence matrix (3D, averaged) | Inverse difference moment | 0,547 | 0,547492 | 0 | 0 |
| configuration C | Co-occurrence matrix (3D, averaged) | Inverse difference moment normalised | 0,994 | 0,994355 | 0 | 0 |
| configuration C | Co-occurrence matrix (3D, averaged) | Inverse variance | 0,39 | 0,39048 | 0 | 0 |
| configuration C | Co-occurrence matrix (3D, averaged) | Correlation | 0,869 | 0,869298 | 0 | 0 |
| configuration C | Co-occurrence matrix (3D, averaged) | Autocorrelation | 1580 | 1583,44 | 0 | 0 |
| configuration C | Co-occurrence matrix (3D, averaged) | Cluster tendency | 276 | 275,803 | 0 | 0 |
| configuration C | Co-occurrence matrix (3D, averaged) | Cluster shade | -10600 | -10616,2 | 0 | 0 |
| configuration C | Co-occurrence matrix (3D, averaged) | Cluster prominence | 569000 | 568750 | 0 | 0 |
| configuration C | Co-occurrence matrix (3D, averaged) | Information correlation 1 | -0,236 | -0,236213 | 0 | 0 |
| configuration C | Co-occurrence matrix (3D, averaged) | Information correlation 2 | 0,9 | 0,899988 | 0 | 0 |
| configuration C | Co-occurrence matrix (3D, merged) | Joint maximum | 0,111 | 0,110929 | 0 | 0 |
| configuration C | Co-occurrence matrix (3D, merged) | Joint average | 39 | 38,9764 | 0 | 0 |
| configuration C | Co-occurrence matrix (3D, merged) | Joint variance | 73,8 | 73,7816 | 0 | 0 |
| configuration C | Co-occurrence matrix (3D, merged) | Joint entropy | 6,42 | 6,41977 | 0 | 0 |
| configuration C | Co-occurrence matrix (3D, merged) | Difference average | 2,16 | 2,16267 | 0 | 0 |
| configuration C | Co-occurrence matrix (3D, merged) | Difference variance | 14,4 | 14,4289 | 0 | 0 |
| configuration C | Co-occurrence matrix (3D, merged) | Difference entropy | 2,64 | 2,64283 | 0 | 0 |
| configuration C | Co-occurrence matrix (3D, merged) | Sum average | 78 | 77,953 | 0 | 0 |
| configuration C | Co-occurrence matrix (3D, merged) | Sum variance | 276 | 276,02 | 0 | 0 |
| configuration C | Co-occurrence matrix (3D, merged) | Sum entropy | 4,56 | 4,5594 | 0 | 0 |
| configuration C | Co-occurrence matrix (3D, merged) | Angular second moment | 0,0447 | 0,044715 | 0 | 0 |
| configuration C | Co-occurrence matrix (3D, merged) | Contrast | 19,1 | 19,1061 | 0 | 0 |
| configuration C | Co-occurrence matrix (3D, merged) | Dissimilarity | 2,16 | 2,16267 | 0 | 0 |
| configuration C | Co-occurrence matrix (3D, merged) | Inverse difference | 0,583 | 0,58275 | 0 | 0 |
| configuration C | Co-occurrence matrix (3D, merged) | Inverse difference normalised | 0,966 | 0,966257 | 0 | 0 |
| configuration C | Co-occurrence matrix (3D, merged) | Inverse difference moment | 0,548 | 0,547883 | 0 | 0 |
| configuration C | Co-occurrence matrix (3D, merged) | Inverse difference moment normalised | 0,994 | 0,994375 | 0 | 0 |
| configuration C | Co-occurrence matrix (3D, merged) | Inverse variance | 0,39 | 0,390464 | 0 | 0 |
| configuration C | Co-occurrence matrix (3D, merged) | Correlation | 0,871 | 0,870524 | 0 | 0 |
| configuration C | Co-occurrence matrix (3D, merged) | Autocorrelation | 1580 | 1583,39 | 0 | 0 |
| configuration C | Co-occurrence matrix (3D, merged) | Cluster tendency | 276 | 276,02 | 0 | 0 |
| configuration C | Co-occurrence matrix (3D, merged) | Cluster shade | -10600 | -10629,8 | 0 | 0 |
| configuration C | Co-occurrence matrix (3D, merged) | Cluster prominence | 570000 | 569595 | 0 | 0 |
| configuration C | Co-occurrence matrix (3D, merged) | Information correlation 1 | -0,228 | -0,228323 | 0 | 0 |
| configuration C | Co-occurrence matrix (3D, merged) | Information correlation 2 | 0,899 | 0,899359 | 0 | 0 |
| configuration C | Run length matrix (3D, averaged) | Short runs emphasis | 0,786 | 0,785948 | 0 | 0 |
| configuration C | Run length matrix (3D, averaged) | Long runs emphasis | 3,31 | 3,3108 | 0 | 0 |
| configuration C | Run length matrix (3D, averaged) | Low grey level run emphasis | 0,00155 | 0,001548 | 0 | 0 |
| configuration C | Run length matrix (3D, averaged) | High grey level run emphasis | 1470 | 1471,64 | 0 | 0 |
| configuration C | Run length matrix (3D, averaged) | Short run low grey level emphasis | 0,00136 | 0,00136 | 0 | 0 |
| configuration C | Run length matrix (3D, averaged) | Short run high grey level emphasis | 1100 | 1097,17 | 0 | 0 |
| configuration C | Run length matrix (3D, averaged) | Long run low grey level emphasis | 0,00317 | 0,003172 | 0 | 0 |
| configuration C | Run length matrix (3D, averaged) | Long run high grey level emphasis | 5590 | 5586,65 | 0 | 0 |
| configuration C | Run length matrix (3D, averaged) | Grey level non-uniformity | 3180 | 3179,1 | 0 | 0 |
| configuration C | Run length matrix (3D, averaged) | Grey level non-uniformity normalised | 0,102 | 0,101571 | 0 | 0 |
| configuration C | Run length matrix (3D, averaged) | Run length non-uniformity | 18000 | 17989,8 | 0 | 0 |
| configuration C | Run length matrix (3D, averaged) | Run length non-uniformity normalised | 0,574 | 0,574353 | 0 | 0 |
| configuration C | Run length matrix (3D, averaged) | Run percentage | 0,679 | 0,679133 | 0 | 0 |
| configuration C | Run length matrix (3D, averaged) | Grey level variance | 101 | 101,45 | 0 | 0 |
| configuration C | Run length matrix (3D, averaged) | Run length variance | 1,12 | 1,12086 | 0 | 0 |
| configuration C | Run length matrix (3D, averaged) | Run entropy | 5,35 | 5,34766 | 0 | 0 |
| configuration C | Run length matrix (3D, merged) | Short runs emphasis | 0,787 | 0,787124 | 0 | 0 |
| configuration C | Run length matrix (3D, merged) | Long runs emphasis | 3,28 | 3,27594 | 0 | 0 |
| configuration C | Run length matrix (3D, merged) | Low grey level run emphasis | 0,00155 | 0,001547 | 0 | 0 |
| configuration C | Run length matrix (3D, merged) | High grey level run emphasis | 1470 | 1472,5 | 0 | 0 |
| configuration C | Run length matrix (3D, merged) | Short run low grey level emphasis | 0,00136 | 0,00136 | 0 | 0 |
| configuration C | Run length matrix (3D, merged) | Short run high grey level emphasis | 1100 | 1099,95 | 0 | 0 |
| configuration C | Run length matrix (3D, merged) | Long run low grey level emphasis | 0,00314 | 0,003144 | 0 | 0 |
| configuration C | Run length matrix (3D, merged) | Long run high grey level emphasis | 5530 | 5525,45 | 0 | 0 |
| configuration C | Run length matrix (3D, merged) | Grey level non-uniformity | 41300 | 41297,7 | 0 | 0 |
| configuration C | Run length matrix (3D, merged) | Grey level non-uniformity normalised | 0,102 | 0,10173 | 0 | 0 |
| configuration C | Run length matrix (3D, merged) | Run length non-uniformity | 234000 | 233618 | 0 | 0 |
| configuration C | Run length matrix (3D, merged) | Run length non-uniformity normalised | 0,575 | 0,57548 | 0 | 0 |
| configuration C | Run length matrix (3D, merged) | Run percentage | 0,679 | 0,679133 | 0 | 0 |
| configuration C | Run length matrix (3D, merged) | Grey level variance | 101 | 101,385 | 0 | 0 |
| configuration C | Run length matrix (3D, merged) | Run length variance | 1,11 | 1,10779 | 0 | 0 |
| configuration C | Run length matrix (3D, merged) | Run entropy | 5,35 | 5,35049 | 0 | 0 |
| configuration C | Size zone matrix (3D) | Small zone emphasis | 0,695 | 0,69499 | 0 | 0 |
| configuration C | Size zone matrix (3D) | Large zone emphasis | 38900 | 38927,2 | 0 | 0 |
| configuration C | Size zone matrix (3D) | Low grey level emphasis | 0,00235 | 0,00235 | 0 | 0 |
| configuration C | Size zone matrix (3D) | High grey level emphasis | 971 | 970,711 | 0 | 0 |
| configuration C | Size zone matrix (3D) | Small zone low grey level emphasis | 0,0016 | 0,001595 | 0 | 0 |
| configuration C | Size zone matrix (3D) | Small zone high grey level emphasis | 657 | 656,828 | 0 | 0 |
| configuration C | Size zone matrix (3D) | Large zone low grey level emphasis | 21,6 | 21,5513 | 0 | 0 |
| configuration C | Size zone matrix (3D) | Large zone high grey level emphasis | 70700000 | 7,07E+07 | 0 | 0 |
| configuration C | Size zone matrix (3D) | Grey level non-uniformity | 195 | 195,032 | 0 | 0 |
| configuration C | Size zone matrix (3D) | Grey level non uniformity normalised | 0,0286 | 0,028643 | 0 | 0 |
| configuration C | Size zone matrix (3D) | Zone size non-uniformity | 3040 | 3042,89 | 0 | 0 |
| configuration C | Size zone matrix (3D) | Zone size non-uniformity normalised | 0,447 | 0,446892 | 0 | 0 |
| configuration C | Size zone matrix (3D) | Zone percentage | 0,148 | 0,148083 | 0 | 0 |
| configuration C | Size zone matrix (3D) | Grey level variance | 106 | 105,968 | 0 | 0 |
| configuration C | Size zone matrix (3D) | Zone size variance | 38900 | 38881,6 | 0 | 0 |
| configuration C | Size zone matrix (3D) | Zone size entropy | 7 | 6,99822 | 0 | 0 |
| configuration C | Distance zone matrix (3D) | Small distance emphasis | 0,531 | 0,553283 | 0,022 | 4,143126 |
| configuration C | Distance zone matrix (3D) | Large distance emphasis | 11 | 11,2992 | 0,2 | 1,818182 |
| configuration C | Distance zone matrix (3D) | Low grey level emphasis | 0,00235 | 0,002408 | 0,00005 | 2,12766 |
| configuration C | Distance zone matrix (3D) | High grey level emphasis | 971 | 966,831 | 4 | 0,411946 |
| configuration C | Distance zone matrix (3D) | Small distance low grey level emphasis | 0,00149 | 0,001576 | 0,00008 | 5,369128 |
| configuration C | Distance zone matrix (3D) | Small distance high grey level emphasis | 476 | 492,975 | 16 | 3,361345 |
| configuration C | Distance zone matrix (3D) | Large distance low grey level emphasis | 0,0154 | 0,016471 | 0,001 | 6,493506 |
| configuration C | Distance zone matrix (3D) | Large distance high grey level emphasis | 13400 | 13637,3 | 200 | 1,492537 |
| configuration C | Distance zone matrix (3D) | Grey level non-uniformity | 195 | 175,675 | 19 | 9,74359 |
| configuration C | Distance zone matrix (3D) | Grey level non-uniformity normalised | 0,0286 | 0,028248 | 0,0003 | 1,048951 |
| configuration C | Distance zone matrix (3D) | Zone distance non-uniformity | 1870 | 1812,33 | 50 | 2,673797 |
| configuration C | Distance zone matrix (3D) | Zone distance non-uniformity normalised | 0,274 | 0,291417 | 0,017 | 6,20438 |
| configuration C | Distance zone matrix (3D) | Zone percentage | 0,148 | 0,135252 | 0,012 | 8,108108 |
| configuration C | Distance zone matrix (3D) | Grey level variance | 106 | 107,519 | 1 | 0,943396 |
| configuration C | Distance zone matrix (3D) | Zone distance variance | 4,6 | 4,94064 | 0,34 | 7,391304 |
| configuration C | Distance zone matrix (3D) | Zone distance entropy | 7,56 | 7,56277 | 0 | 0 |
| configuration C | Neighbourhood grey tone difference matrix (3D) | Coarseness | 0,000216 | 0,000216 | 0 | 0 |
| configuration C | Neighbourhood grey tone difference matrix (3D) | Contrast | 0,0873 | 0,087314 | 0 | 0 |
| configuration C | Neighbourhood grey tone difference matrix (3D) | Busyness | 1,39 | 1,39119 | 0 | 0 |
| configuration C | Neighbourhood grey tone difference matrix (3D) | Complexity | 1810 | 1807,93 | 0 | 0 |
| configuration C | Neighbourhood grey tone difference matrix (3D) | Strength | 0,651 | 0,649926 | 0,001 | 0,15361 |
| configuration C | Neighbouring grey level dependence matrix (3D) | Low dependence emphasis | 0,137 | 0,136901 | 0 | 0 |
| configuration C | Neighbouring grey level dependence matrix (3D) | High dependence emphasis | 126 | 126,491 | 0 | 0 |
| configuration C | Neighbouring grey level dependence matrix (3D) | Low grey level count emphasis | 0,0013 | 0,001297 | 0 | 0 |
| configuration C | Neighbouring grey level dependence matrix (3D) | High grey level count emphasis | 1570 | 1567,86 | 0 | 0 |
| configuration C | Neighbouring grey level dependence matrix (3D) | Low dependence low grey level emphasis | 0,000306 | 0,000306 | 0 | 0 |
| configuration C | Neighbouring grey level dependence matrix (3D) | Low dependence high grey level emphasis | 141 | 140,581 | 0 | 0 |
| configuration C | Neighbouring grey level dependence matrix (3D) | High dependence low grey level emphasis | 0,0828 | 0,082808 | 0 | 0 |
| configuration C | Neighbouring grey level dependence matrix (3D) | High dependence high grey level emphasis | 227000 | 226736 | 0 | 0 |
| configuration C | Neighbouring grey level dependence matrix (3D) | Grey level non-uniformity | 6420 | 6416,53 | 0 | 0 |
| configuration C | Neighbouring grey level dependence matrix (3D) | Grey level non-uniformity normalised | 0,14 | 0,139547 | 0 | 0 |
| configuration C | Neighbouring grey level dependence matrix (3D) | Dependence count non-uniformity | 2450 | 2447,44 | 0 | 0 |
| configuration C | Neighbouring grey level dependence matrix (3D) | Dependence count non-uniformity normalised | 0,0532 | 0,053227 | 0 | 0 |
| configuration C | Neighbouring grey level dependence matrix (3D) | Dependence count percentage | 1 | 1 | 0 | 0 |
| configuration C | Neighbouring grey level dependence matrix (3D) | Grey level variance | 81,1 | 81,1122 | 0 | 0 |
| configuration C | Neighbouring grey level dependence matrix (3D) | Dependence count variance | 39,2 | 39,2081 | 0 | 0 |
| configuration C | Neighbouring grey level dependence matrix (3D) | Dependence count entropy | 7,54 | 7,53669 | 0 | 0 |
| configuration C | Neighbouring grey level dependence matrix (3D) | Dependence count energy | 0,00789 | 0,007891 | 0 | 0 |
